# Sources of the N15 TMS-evoked potential following motor cortex stimulation localize rostrals to TMS-induced electric fields and depend on dose

**DOI:** 10.1162/IMAG.a.1356

**Published:** 2026-09-10

**Authors:** Sybren Van Hoornweder, Mikkel M. Beck, Jesper D. Nielsen, Leo Tomasevic, Raf L.J. Meesen, Hartwig R. Siebner, Axel Thielscher

**Affiliations:** Danish Research Centre for Magnetic Resonance, Centre for Functional and Diagnostic Imaging and Research, Copenhagen University Hospital Amager and Hvidovre, Hvidovre, Denmark; REVAL - Rehabilitation Research Center, Faculty of Rehabilitation Sciences, University of Hasselt, Diepenbeek, Belgium; Department of Psychiatry and Psychotherapy, University of Regensburg, Regensburg, Germany; Department of Human Sciences, Institute of Psychology, University of the Bundeswehr Munich, Neubiberg, Germany; Movement Control and Neuroplasticity Research Group - Department of Movement Sciences, Group Biomedical Sciences, KU Leuven, Leuven, Belgium; Department of Neurology, Copenhagen University Hospital Bispebjerg, Copenhagen, Denmark; Institute for Clinical Medicine, Faculty of Medical and Health Sciences, University of Copenhagen, Copenhagen, Denmark; Department of Health Technology, Technical University of Denmark, Kgs. Lyngby, Denmark

**Keywords:** TMS, EEG, N15, source modeling, electric fields, dose, state, TEP

## Abstract

Transcranial magnetic stimulation (TMS) elicits characteristic cortical responses known as TMS-evoked potentials (TEPs) measured via electroencephalography (EEG). An early negative peak can be consistently evoked over the motor cortex at around 15 ms (N15). It remains elusive whether N15 overlaps with the cortical regions receiving the highest TMS-induced electric field (E-field). We characterized the source activity of the N15 component relative to the TMS-induced E-field in 16 healthy adults who underwent TMS-EEG over the left primary motor hand area at six stimulus intensities (-8% to +12% maximum stimulator output relative to resting motor threshold) during rest and tonic contraction. Individualized head models, based on T1 and T2 MRI scans, were used for E-field simulations and source localization with LCMV, dSPM, and eLORETA as inverse solvers. Linear mixed-effects models and spatial clustering were used to assess dose- and state-dependent effects. The N15 source field was consistently located rostral to the TMS-induced electric field, with the spatial disparity decreasing as the stimulation dose increased. N15 source activity primarily clustered in the left premotor and primary motor cortex, while the TMS E-field peaked in the crowns of the pre- and postcentral gyri. All three inverse solvers yielded similar results, demonstrating consistent dose- and state-dependent effects on the N15 and suggesting that solver choice had minimal impact. These findings demonstrate a spatial mismatch between early TMS-evoked potentials following motor cortex stimulation and the cortical regions exposed to peak electric fields. Combined with prior literature, our findings provide evidence that the rostral N15 source activity reflects transsynaptic propagation via direct cortico-cortical or indirect cortico-subcortico-cortical pathways.

## 1 Introduction

Transcranial magnetic stimulation (TMS) coupled with electroencephalography (EEG) has emerged as a powerful tool in basic, cognitive, and clinical neuroscience (Cao et al., 2021; Chung et al., 2015; Farzan & Bortoletto, 2022; Kallioniemi & Daskalakis, 2022; Tremblay et al., 2019; Ziemann et al., 2026). By directly stimulating the cortex via TMS and registering the neural responses via EEG, TMS-EEG offers a unique noninvasive neurophysiological measure via the TMS-evoked potential (TEP).

TEPs are a series of voltage deflections phase-locked to the TMS-pulse that reflect the cortical response to the TMS-induced electric field within the first few hundred milliseconds. Each TEP deflection represents distinct neurophysiological processes originating from various cortical regions (Ahn & Fröhlich, 2021; Chung et al., 2015; Farzan & Bortoletto, 2022; Farzan et al., 2016; Nuyts, Chalavi, et al., 2026; Rogasch & Fitzgerald, 2013). One of the earliest deflections obtained when stimulating the primary motor cortex (M1) is termed N15, a negative voltage peak occurring at a latency of ~10 to 15 ms. While its short latency leads to some methodological challenges (Farzan & Bortoletto, 2022), it also positions N15 as an indicator of relatively localized cortical responsiveness.

However, the origin of N15 remains subject to debate. It is unclear whether N15 reflects direct or indirect activation from the same neural subpopulations that were the primary recipients of the TMS-induced electric field, or from distinct neuronal subpopulations, and if so, which. Studies have pinpointed N15 to several regions, among which the ipsilateral M1, premotor cortex, supplementary motor area and posterior parietal cortex (Bonato et al., 2006; Chowdhury et al., 2023; Esser et al., 2006; Ferreri et al., 2011; Litvak et al., 2007; Mäki & Ilmoniemi, 2010; Rawji et al., 2021; Zazio et al., 2021). These discrepancies in localization may partly be due to differences in TMS paradigms, such as the use of sub- vs. suprathreshold intensities (e.g., Ahn & Fröhlich, 2021; Bestmann et al., 2004). However, they likely also stem from anatomically suboptimal forward models and varying inverse solvers, as these factors affect source localization accuracy (Chowdhury et al., 2023; Esser et al., 2006; Grech et al., 2008; Halder et al., 2019; Litvak et al., 2007; Mäki & Ilmoniemi, 2010; Nielsen et al., 2023; Zazio et al., 2021).

A direct spatial comparison between the N15 source and the TMS-induced electric field is lacking. While the TMS-induced electric field is not identical to the effectively stimulated region, it does provide an indication of where direct stimulation effects are most likely to occur, with peak TMS-induced electric fields for M1 stimulation broadly overlapping with the assumed primary cortical activation sites of M1 TMS (Siebner et al., 2022). Comparing the TMS electric field and N15 source field could provide valuable insights into whether N15 represents reactivation of the same neuronal populations that were most exposed to TMS, or the engagement of other cortical circuitry.

Here, we bridged this gap by employing anatomically accurate forward models (Nielsen et al., 2023) and comparing three inverse solvers: linearly constrained minimum variance beamforming (LCMV) (Veen et al., 1997; Westner et al., 2022), dynamic statistical parametric mapping (dSPM) (Dale et al., 2000), and exact low resolution brain electromagnetic tomography (eLORETA) (Pascual-Marqui et al., 2011). Building on previous findings from our group which demonstrated that the N15 generated by TMS over M1 differs fundamentally from motor evoked potentials (MEPs) in its response to motor state but yields similar responses to changes in dose, we aimed to further characterize the neural origins and functional properties of N15 (Beck et al., 2025). Specifically, our primary objective was to compare the spatial origin of N15 with the preceding TMS-induced electric field, while considering variations in dose and brain state. Additionally, we sought to replicate prior dose- and state-dependent results at the N15 source level and qualitatively and quantitatively compare the three aforementioned inverse solvers.

We had no prior hypotheses for the spatial concordance analyses given the exploratory nature of the current work, and the partly contradicting prior literature (Bonato et al., 2006; Chowdhury et al., 2023; Esser et al., 2006; Ferreri et al., 2011; Litvak et al., 2007; Mäki & Ilmoniemi, 2010; Rawji et al., 2021; Zazio et al., 2021). Likewise, we expected all three inverse solvers to yield similar results. Informed by our previous sensor-level analyses of these data (Beck et al., 2025), we expected N15 source activity to depend on state and dose.

## 2 Methods

### 2.1 Participants

We aimed for a sample size in the upper range of those used in earlier TMS-EEG studies examining N15 (ranging from n = 5 to 28, mean n = 12; Chowdhury et al., 2023; Esser et al., 2006; Litvak et al., 2007; Mäki & Ilmoniemi, 2010; Zazio et al., 2021). Following exclusion of 12 participants due to the inability to obtain TEPs without muscle artefacts, 16 of 28 screened healthy younger adults were enrolled (11 women, mean age = 27 ± 3 years old [mean ± standard deviation]). Based on the Edinburgh Handedness Inventory, 14 out of 16 participants were right-handed (Oldfield, 1971). Participants had no known history of neurological and/or psychiatric disorders, or TMS contraindications (Rossi et al., 2009). Written informed consent was obtained, and the study adhered to the Declaration of Helsinki, being approved by the Ethical Committee of the Capital Region of Denmark (H-15008824). One participant did not undergo TMS at the intensity of rMT + 12% maximal stimulator output (MSO) due to discomfort. Also, in 4 participants, 1 out of 12 conditions was removed post-EEG preprocessing due to insufficient remaining epochs (<30).

### 2.2 Experimental procedures and data acquisition

**Fig. 1.**
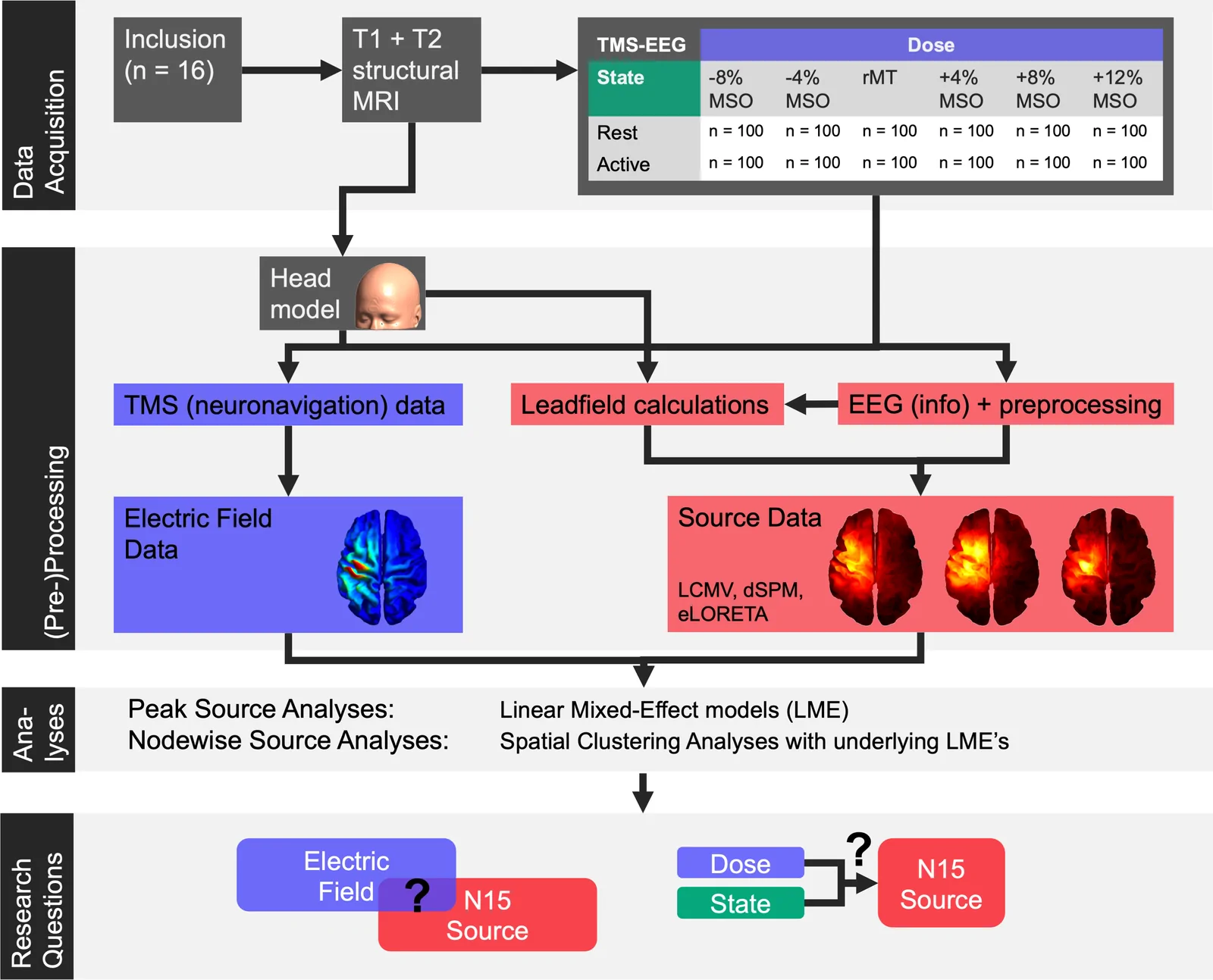
Experimental workflow. After inclusion, MRI scans were obtained and TMS-EEG was performed. The data were (pre-)processed and analyzed to address two questions per inverse solver: (1) Are the N15 source- and TMS electric field spatially concordant? (2) Does the N15 source activity depend on motor state- and/or TMS dose?

An overview of this within-subject, repeated-measures design is shown in Figure 1. Following inclusion, T1- and T2-weighted structural MRI scans were acquired. Then, neuronavigated TMS-EEG was performed at six doses and two motor states, resulting in 12 conditions. Participants were instructed to keep their eyes open, avoid blinking, and fixate their gaze at a spot ~1 m anterior to the participant. EEG, neuronavigation, and MRI data were (pre-)processed and analyzed to address our research questions.

#### 2.2.1 Structural MRI

T1- and T2-weighted structural MRI scans were acquired with a 3T Prisma scanner (Siemens Healthineers AG, Forcheim, Germany) for EEG source modeling and TMS electric field modeling. T1 scans had an isotropic resolution of voxel size = 0.9 mm^3^ (TR = 2,700 ms, TE = 3.7 ms, flip angle = 9°), and T2 scans had an isotropic resolution of voxel size = 0.9 mm^3^ (TR = 3,200 ms, TE = 408 ms, flip angle = 120°).

#### 2.2.2 TMS-EEG and EMG

TMS was applied with the MagVenture MC-B70 figure-of-eight coil and X100 stimulator. Biphasic antero-posterior postero-anterior pulses were administered with an interstimulus interval of 2 ± 0.2 seconds jitter. The stimulator recharge was delayed to 500 ms. Neuronavigation (Localite GmbH, Bonn, Germany) tracked the coil position relative to each participant’s T1 scan. A belly-tendon EMG set-up registered MEPs of the right first dorsal interosseus muscle (FDI), with the ground positioned on the right ulnar styloid process. EMG recordings were amplified (x500), bandpass filtered (2–2000 Hz), and sampled at 2 kHz (Digitimer D360, Digitimer Ltd., Hertfordshire, UK) via Signal software (v4.17, Cambridge Electronic Design, Cambridge, UK).

A functional hotspot search was done to identify the location where the largest most consistent FDI motor evoked potential could be elicited. The anatomical location of the hand-knob was used as starting point, and hotspotting was done with the coil handle at a ~45° angle to the mid-sagittal plane. The hotspot was digitized using neuronavigation, and the rMT was acquired via the ML-PEST approach (Awiszus, 2003; Awiszus et al., 1999). Next, we assessed if cranial MEPs contaminated the EEG data during TMS over the hotspot. If so, minor adjustments to the coil tilt, angle, and position were made to steer away from the cranial muscles. This new coil position, which differed only slightly from the original position, was digitized via neuronavigation as the final hotspot and used for TMS administration throughout the experiment and for TMS electric field modeling (cf., Section 2.3.2). As aforementioned, in 12 out of 28 participants, no clean, muscle-artefact-free, TEPs could be achieved, resulting in their exclusion.

Twelve blocks differing in terms of state and/or dose were acquired, for a total of 1,200 TMS pulses (6 [doses] * 2 [states] * 100 [TMS pulses]; Fig. 1). Concerning state, active (i.e., 10% maximum voluntary contraction of the FDI) and resting state (FDI at rest) were distinguished. Concerning dose, TMS intensities were scaled based on the rMT, ranging from rMT-8% MSO to rMT+12% MSO in 4% increments.

EEG data were recorded at 5 kHz from 61 passive Ag/AgCl electrodes (M10 layout, BrainCap TMS, Brain Products GmbH, Germany), using the actiChamp Plus64 system (Brain Products GmbH, Germany), and stored on a personal computer for offline analyses. The ground and reference electrodes were placed on the right and left sides of the participant’s forehead. All electrodes were prepared using electroconductive abrasive gel to reduce impedances < 5 kΩ.

Throughout the study, participants wore modified earplugs playing pink noise with recorded TMS clicks, generated via TMS Adaptable Auditory Control (Russo et al., 2022). A ~1.5 mm thick foam layer was placed on the active TMS coil side to minimize tactile and auditory co-stimulation. Upon TMS pulse application, a trigger was sent to the EEG device.

### 2.3 Data (pre-)processing

#### 2.3.1 TMS: Electric field modeling

To model the TMS-induced electric fields, the T1 and T2-weighted scans were segmented and meshed into volume conductor models of the head via SimNIBS-CHARM (v.4.0.1) (Puonti, Van Leemput, et al., 2020), for use in Finite-Element Method (FEM) simulations. In one participant, only the T1 scan was used. The resultant head meshes were visually examined for anatomical accuracy. Standard SimNIBS conductivity values were used (Supplementary Table S1.1), and the neuronavigation-digitized TMS coil position was imported.

In nine participants, the neuronavigation data, and specifically the x, y, and z coordinates of the center coil position, were corrupt. To enable electric field simulations in these participants, the correct TMS coil coordinates of the remaining participants were warped to MNI space, averaged, and then warped back to the subject space. Supplementary Materials S3 provides several control analyses, including a leave-one-out analysis in the participants with accurate data (Supplementary Figs. S3.1 and S3.2) that indicates that the peak electric field magnitude (|E|) centroid in the accurate vs. interpolated data differed by 2.88 mm on average. This, alongside other control analyses (Supplementary Figs. S3.3. and S3.4.), suggests that this approach was effective both in terms of ensuring accurate field magnitudes and spatial field distributions. Also, our results in Section 3.1 indicate that electric field magnitude, relative intensity, and stimulator output all performed similarly in predicting N15 source magnitude, further suggesting that our interpolation method was effective.

The speed of current variation through the TMS coil (dI/dt, A/s) was calculated as

$$\frac{dI}{dt}=\%MSO\times 151e^{6}$$ (1)

The resultant |E| simulations were warped to fsaverage5 space (20,484 nodes) to allow for group-level analyses and comparisons with the EEG source data.

#### 2.3.2 EEG: Preprocessing

**Fig. 2.**
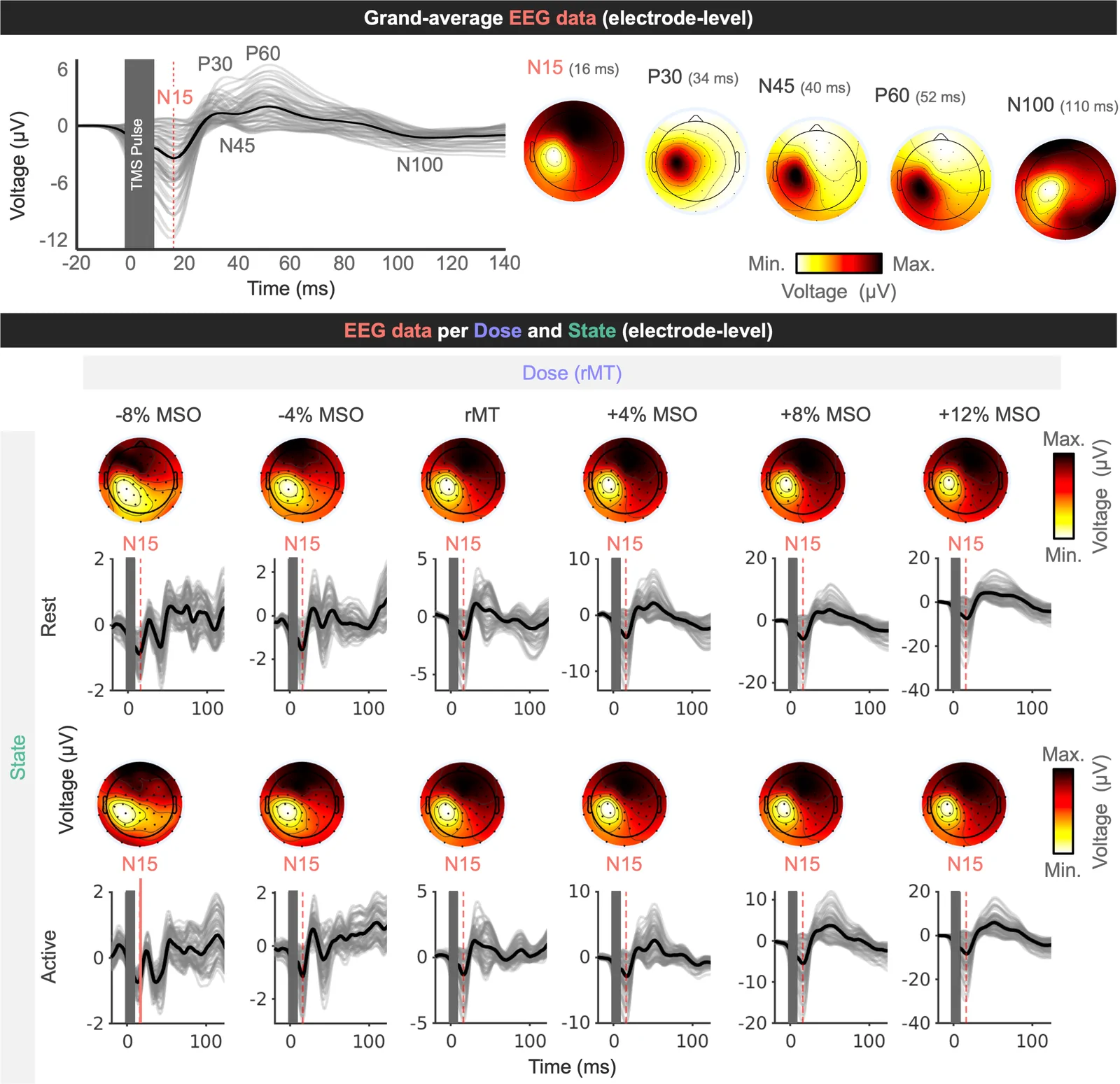
Group-level sensor-space data. The top panel shows the group-average across all states and doses. On the left, the butterfly plot shows the voltage of all channels (grey) and the mean (block) over time. The TMS-pulse was administered at 0 ms. The grey rectangular area shows the interpolation window. On the right, the topographies of five prototypical TEP components, including N15, are shown. The lower panel shows the group-level butterfly plot and N15 topography per condition, and state Supplementary Figures S2.3 and S2.4 show the individual participant-level data.

EEG data were preprocessed in MATLAB (v2022b, The MathWorks Inc., Portola Valley, CA, USA) using custom-scripts based on EEGLAB (Delorme & Makeig, 2004) and TESA (Rogasch et al., 2017). Channel info was added from a custom channel lay-out file (Nielsen et al., 2023). Epochs spanning from -600 to 600 ms were extracted, with 0 ms being the TMS pulse administration. Data between -2 to 8 ms, containing the TMS pulse artefact, were removed and interpolated via cubic interpolation, with a cubic fitting window of -20 to 20 ms. The epochs were merged into a continuous dataset, filtered using a 1 – 48 Hz 2^nd^ order Butterworth filter, re-epoched into -500 to 500 ms epochs, and visually checked for bad channels, resulting in the removal of 5 ± 3 channels per dataset (mean ± standard deviation) (range: 2 – 13 channels) (cf., Supplementary Fig. S2.1 for more info on the removed channels). Missing channels were interpolated, and noisy epochs and epochs containing eyeblinks were manually rejected, resulting in the removal of 14 ± 11 epochs per condition (range: 0–54 rejected epochs out of 100 epochs per condition). Finally, EEG data were baseline corrected (-110 to -10 ms window); the data were downsampled to 500 Hz and re-referenced to the common average reference. Our preprocessing pipeline did not apply any artefact suppression methods (e.g., ICA) in line with Beck, Heyl, et al. (2024). Instead, we adopted a conservative approach based on stringent visual inspection and exclusion of participants exhibiting signs of muscle artefacts. The effectiveness of our approach is supported by group-level visualizations (Fig. 2), as well as participant-level butterfly plots and topographical maps (Supplementary Figs. S2.3 and S2.4).

#### 2.3.3 EEG: Source modeling

EEG Source modeling was done in Python (v3.11.4) via MNE (v1.5.1) (Gramfort et al., 2013) and SimNIBS (v.4.0.1) (Nielsen et al., 2023; Puonti, Van Leemput, et al., 2020; Thielscher et al., 2015). Initially, the EEG channels were co-registered to each participants head model (cf., Section 2.3.1). The forward model was created in line with Nielsen et al. (2023). This involved using the head model obtained through CHARM, employing the same tissue conductivities as used for TMS simulations, integrating co-registered EEG positions, and defining a source space corresponding to the middle grey matter surface.

Next, the noise and data covariance matrices were computed using data from -110 to -10 ms and 10 to 115 ms, respectively. The data rank was determined via eigenvalue indices, and covariances were calculated using the shrunk method (Engemann & Gramfort, 2015). Four inverse solvers were applied: (1) LCMV, (2) dSPM, (3) eLORETA, and (4) minimum-norm estimation (Ou et al., 2009). The first three are our primary focus, minimum-norm estimation is included in Supplementary Figure S2.2, in line with Ahn & Fröhlich (2021). Standard MNE functions were used for all solvers. Per condition and participant, all epochs were first averaged. For LCMV, a default regularization value of 0.05 was used. The orientation was optimized to maximize output power, and unit-noise-gain weighting was applied to address depth bias (Gramfort et al., 2013; Veen et al., 1997; Westner et al., 2022).

For minimum-norm-estimation, dSPM and eLORETA, default values for depth weighting (d = 2) (Lin et al., 2006), signal-to-noise (STN = 3), and dipole orientation (loose = 0.2) were used.

All source fields were warped onto the fsaverage5 template to enable group analyses and comparisons with the TMS electric fields. Statistical analyses of source data were conducted at timepoint = 16 ms, with analyses for 14 ms yielding identical results.

Notably, while the shared preprocessing and forward-modeling steps across inverse solvers enable controlled comparisons, they may also introduce common biases, meaning that our analyses do not assess each solver’s sensitivity to variations in these assumptions.

### 2.4 Statistical analyses

Peak source magnitude, |S|, analyses, with peak activity defined as the 99.9^th^ percentile source magnitude, were performed in R (v.4.3.1, R Foundation for Statistical Computing, Vienna, Austria), using RStudio (ggplot2 [v3.4.2], lme4 [v 1.1.35.1], MuMIn [v1.47.5], effectsize [V0.8.6]) (Barton, 2009; Bates et al., 2015; Ben Shachar et al., 2020; R Core Team, 2021; RStudio Team, 2020; Valero-Mora, 2010). The focus on source magnitude was a deliberate choice, aligning with our conceptual emphasis on electric field magnitude. The significance level was set to α = 0.05. Backward model building was done for all models. Effect sizes were estimated as partial η^2^ where possible to help interpretation with η^2^ > 0.01, 0.06, and 0.14 indicating small, medium and large effect sizes.

Whole-cortex source analyses were done in MATLAB, with α = 0.01. When significant fixed effects were present requiring pairwise comparisons for interpretation, the associated p-values were Tukey-corrected.

All statistical analyses were performed for each inverse solver separately.

#### 2.4.1 Dose quantification: Peak electric field magnitude, absolute or relative stimulator output

Prior to answering our two research questions (cf., Fig. 1), we compared 3 linear mixed effects (LME) models that used alternative methods of defining stimulation dose:

$$\begin{array}{l} \log(|S{|}_{P99.9i,j})=\beta_{0}+\beta_{1}State_{ij}+\beta_{2}|E{|}_{P99.9ij}\\ \text{ +}\beta_{3}(State_{ij}\times |E{|}_{P99.9ij})+b_{0i}+\varepsilon_{i,j}, \end{array}$$ (2a)

$$\begin{array}{l} \log(|S{|}_{P99.9i,j})=\beta_{0}+\beta_{1}State_{ij}+\beta_{2}{\text{\%MSO}}_{ij}\\ \text{ +}\beta_{3}(State_{ij}\times \%MSO_{ij})+b_{0i}+\varepsilon_{i,j}, \end{array}$$ (2b)

$$\begin{array}{l} \log(|S{|}_{P99.9i,j})=\beta_{0}+\beta_{1}State_{ij}+\beta_{2}\text{rMT}\pm {\text{\%MSO}}_{ij}\\ \text{ +}\beta_{3}(State_{ij}\times rMT\pm \%MSO_{ij})+b_{0i}+\varepsilon_{i,j}, \end{array}$$ (2c)

where $\beta_{0}$ is the fixed intercept, and $\beta_{1}$, $\beta_{2}$, and $\beta_{3}$ are the fixed coefficients for dose, state, and their interaction, respectively. Dose is continuous for Equations 2a and 2b. For Equation 2a, dose is represented as the peak electric field magnitude [|E|], quantified as |E|_P99.9_ (Van Hoornweder et al., 2023). For Equation 2b, it is represented as the absolute %MSO of the TMS stimulator. For Equation 2c, dose is categorical, containing six levels from rMT-8%MSO up to rMT+12%MSO. Across all models, state is a categorical variable of two levels: REST or ACTIVE. $b_{0i}$ is the random intercept for each level of participant, and $\varepsilon_{i,j}$ is the residual error. Consistent for all three models and all subsequent analyses, |S| data was log-transformed to adhere to the linearity assumption. The models were compared via their AIC, BIC and R^2^_m_ values, and we aimed to use the most-predictive dose definition in the remainder of this work.

As discussed in Section 3.1, the three dose definitions did not differ much, and dose was quantified as |E|_P99.9_ as this metric was conceptually preferred in light of our study aims. As highlighted in Supplementary Figure S2.6, the absence of substantial differences does not imply general interchangeability of dose definitions, but instead appears specific to the present statistical design and modeling context.

#### 2.4.2 Spatial concordance between electric- and source fields

To achieve our primary aim of demarcating the N15 source distribution with respect to the TMS induced electric field, we examined dose- and state-dependent differences in the relative magnitudes of |S| and |E| across the whole cortex.

First, per participant, dose and state, we quantified the nodewise normalized N15 source magnitude and electric field magnitude via min-max scaling ([data node – minimum data] / [maximum data – minimum data], with data being either the N15 source magnitude or electric field magnitude). Next, permutation-based, spatial clustering analyses were done to assess whole-cortex differences. The main framework of these analyses was based on ACES (Baetens et al., 2024), with two differences being that an LME was used to derive nodewise t-values and that a threshold-free clustering enhancement (TFCE) approach was used (see Maris & Oostenveld, 2007; Smith & Nichols, 2009 for details). Per node, the following linear mixed effect model was run,

$$\begin{array}{l} {\left(|E|,|S|\right)}_{node}=\beta_{0}+\beta_{1}+State_{ij}+\beta_{2}|E{|}_{P99.9\;ij}+\beta_{3}Type_{ij}\\ \text{ +}\beta_{4}(State_{ij}\times |E{|}_{P99.9\;ij})+\beta_{5}+(State_{ij}\times Type_{ij})\\ \text{ +}\beta_{6}(|E{|}_{P99.9\;ij}\times Type_{ij})+b_{0i}+\varepsilon_{i,j}, \end{array}$$ (3)

where Type is a categorical variable (|E| or |S|), ${\left|E\right|}_{P99.9}$ represents the TMS dose, and state refers to the motor state (active vs. rest).

Then, per node and for each fixed effect, the t-ratio was stored and TFCE values were calculated as follows,

$$TFCE_{node}=\int_{h}e{\left(h\right)}^{E}h^{H}dh,$$ (4)

where *h* is cluster height—the t-value cut-off that is used for inclusion per iteration—, and *e* is cluster extent—the number of adjacent nodes exceeding *h*, with adjacency being defined as sharing a side of a triangle making up the grey matter surface. *E* and *H* are the weights for *e* and *h*, respectively, defaulting to *E* = 0.5 and *H* = 2. The increase of *h* per iteration was set the *h_onset_ =* maximum*(t-value)/*2 and the step size to 0.5. As t-values can be negative or positive, and the TFCE approach only considers positive signs, this procedure was repeated with inverted t-value signs. The resultant positive and negative TFCE-values were then combined, after re-adding the original signs.

To obtain a surrogate null-distribution and consequently derive p-values, 800 permutations were performed. Per permutation, the dependent variable was shuffled within each node while maintaining fixed-effect labels. The TFCE procedure was then repeated, resulting in 800 permuted TFCE values per node. TFCE values of the true dataset were considered significant if in the <0.5^th^ or >99.5^th^ percentile of the surrogate null-distribution.

#### 2.4.3 State- and dose-dependency of the N15 source component

To achieve our second aim of replicating the sensor-level state and dose effects on the N15 source level (Beck et al., 2025), we assessed the effect of dose (quantified as |E|_P99.9_) and state on peak source magnitude, |S|_P99.9_, and whole-cortex |S|, per inverse solver.

Effects on peak source magnitude, |S|_P99.9_, were investigated for each inverse solver separately, using the same model as described in Equation 2a.

Whole-cortex state and dose effects on |S| were examined through permutation-based, spatial clustering analyses, similar to Section 2.4.2. Per node of the middle grey matter surface, an LME was fitted:

$$\begin{array}{l} \log(|S{|}_{node\;ij})=\beta_{0}+\beta_{1}State_{ij}+\beta_{2}|E{|}_{P99.9\;ij}\\ \text{ +}\beta_{3}+(State_{ij}\times |E{|}_{P99.9\;ij})+b_{0,i}+\varepsilon_{i,j} \end{array}$$ (5)

Then, per node and for each fixed effect, the t-ratio was stored and TFCE values were calculated (cf. Eq. 4). A surrogate null-distribution was derived through 800 permutations, and TFCE values of the true dataset were considered significant when in the < 0.5^th^ or >99.5^th^ percentile of the surrogate null-distribution.

## 3 Results

Before discussing our main findings, Figure 2 provides an overview of the sensor-level TEP data, including group-average butterfly- and topographic plots. Participant-level data are included in Supplementary Figures S2.3 and S2.4.

### 3.1 Absolute MSO, relative MSO, and electric field magnitude perform similar for peak N15 source magnitude prediction

Before answering our research questions, we compared three approaches to quantify dose in light of peak N15 source activity, |S|_P99.9_: (1) peak electric field magnitude |E|_P99.9_, (2) absolute MSO, and (3) %MSO relative to rMT.

**Fig. 3.**
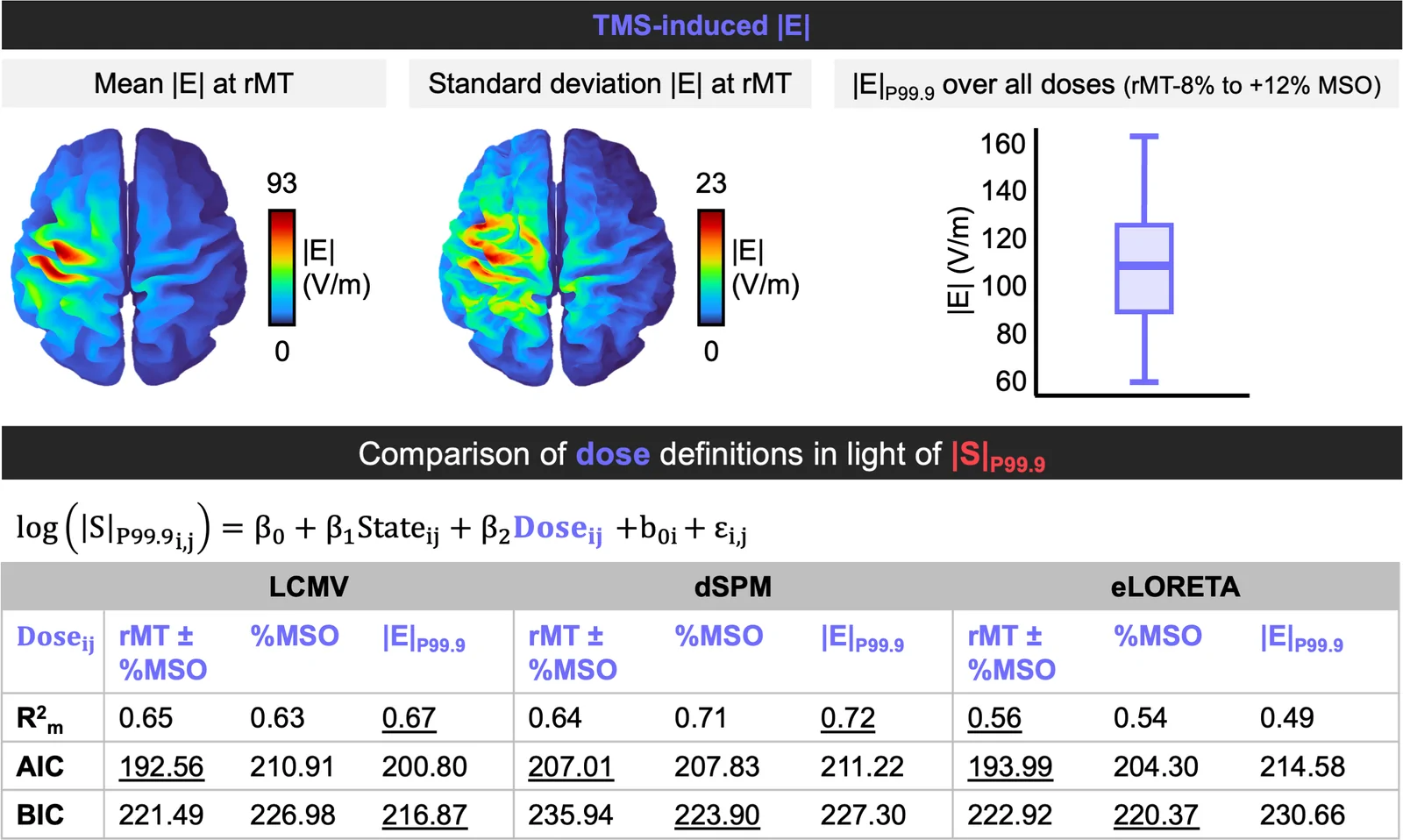
TMS-induced electric field magnitude (|E|) and comparison of three dose definitions (relative: rMT ± %MSO, absolute: %MSO, and |E|: |E|_P99.9_) to predict peak source magnitude (|S|_P99.9_). The upper panel shows the mean and standard deviation of |E| induced at the rMT (left) and |E|_P99.9_ across all doses (right). The lower table compares the three approximations in terms of predicting |S|_P99.9_ per inverse solver via marginal R^2^, AIC and BIC. Per metric and inverse solver, underlined values indicate the best fitting model. The three dose definitions performed similar within our experimental design (cf., Supplementary Figs. S2.5 and S2.6 for additional information on how these dose definitions compare).

Across stimulation intensities, |E|_P99.9_ (Fig. 3, upper panel) values ranged from 58.3 V/m (rMT-8% MSO) to 164.2 V/m (rMT+12% MSO). The mean |E| induced at the rMT intensity was 103.24 ± 14.13 V/m, consistent with prior work (cf., Numssen et al., 2023 for an overview). %MSO values ranged from 20 (rMT–8% MSO) to 52% MSO (rMT+12% MSO), with the mean %MSO at rMT being 35.06 ± 3.66 %MSO. In terms of electric field magnitudes, we observed peak values in the pre- and post-central gyri. This concurs with consensus activation sites of TMS targeting hand muscle representations (i.e., the precentral crown and/or the sulcal wall) (Siebner et al., 2022), with prior work favoring superficial stimulation positions (i.e., the crown) (Bungert et al., 2017; Laakso et al., 2018; Numssen et al., 2021; Weise et al., 2020).

The different dose definitions did not differ much in terms of marginal R^2^, AIC and BIC values for N15 |S|_P99.9_ prediction (Fig. 3, lower table). Depending on the metric, one definition slightly outperformed the others but no consistent pattern emerged, nor were the differences large. In Supplementary Figure S2.5, we directly compare the three dose definitions to each other, further confirming their similarity for our aim of probing within-subject dose effects. Due to these negligible differences, we continued with |E|_P99.9_ for the remainder of the current work given our study aims.

Bigger differences prevailed across solvers. Whereas LCMV and dSPM performed similarly, reaching an R^2^_m_ of ~0.70 at best when evaluating the effects of state and dose on N15 |S|_P99.9_, eLORETA had an R^2^_m_ of 0.56 at best. Thus, source activity reconstructed with LCMV and dSPM seemed to better represent state and dose effects. The specific effects of state and dose are discussed in Section 3.3.

### 3.2 The N15 source field is situated rostral to the TMS-induced electric field, and its location depends on dose

**Fig. 4.**
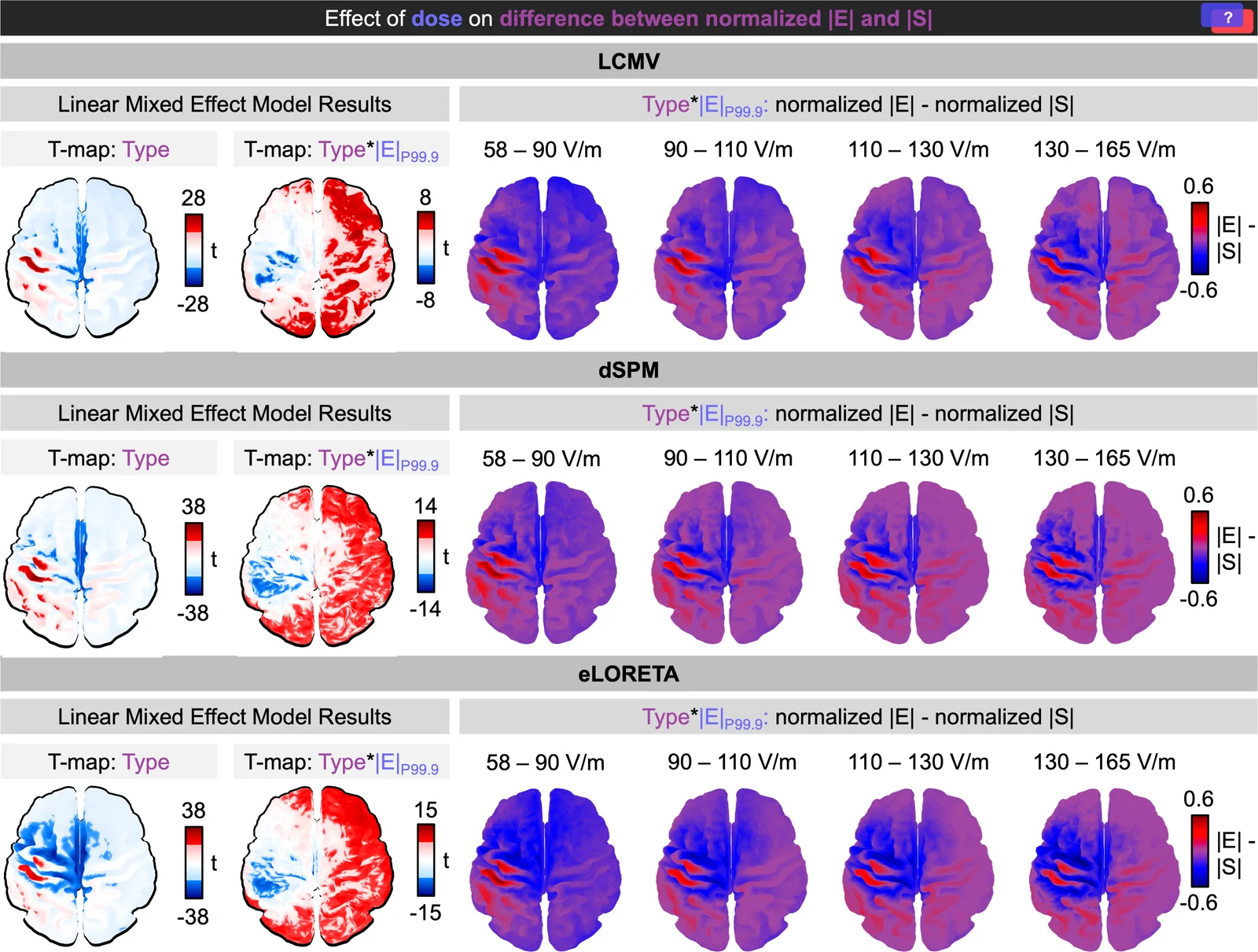
Spatial concordance between the TMS electric field (|E|) and N15 source field (|S|), per inverse solver. The left panel shows the spatial clustering results, regions in clear blue and red were significant. The right panel shows the normalized |E| - |S| difference maps. |E| is larger in the pre- and post-central gyral crowns, while |S| is larger in the premotor regions. Higher doses reduce the relative difference between |E| and |S| in the crown of the precentral gyrus.

Our primary objective was to explore the spatial relationship between the N15 source field, generated by TMS over M1, and the underlying TMS-induced electric field. Spatial clustering analyses conducted across all three solvers revealed significant main effects of field type (electric, |E|, field versus source, |S|, field) and a significant interaction between field type and dose, defined as |E|_P99.9_ (Fig. 4). These findings highlight the distinct spatial patterns between the N15 source field and the TMS-induced electric field. The N15 source field is typically located rostral to the TMS-induced electric fields, which peak in the crowns of the pre- and post-central gyri, in and near the hand knob. This spatial disparity, which is influenced by the TMS dose, indicates that the neural subpopulations generating the N15 response following M1 stimulation do not entirely overlap with those targeted by TMS.

When dose increased, the spatial disparity between the TMS-induced electric field and the N15 source field changed in a region-specific manner. Since the normalized distribution of the electric field remains constant across TMS intensities, the significant interaction between field type and dose reflects a dose-dependent spatial shift of the N15 source fields (Fig. 4, right). When lower TMS doses are used, the electric and source field are most dissimilar in terms of relative spatial presence. As TMS intensity ramps up, the spatial concordance between the N15 source field and the electric field increases. At low doses, the crowns of the pre- and post-central gyri predominantly contain the electric field. However, at higher doses, the relative presence of the electric field diminishes, and N15 source magnitude increases in regions closer to the primary motor cortex hand knob, whereas it decreases in other regions.

This dose-dependent shift in the spatial overlap between the fields may be interpreted in two non-mutually exclusive ways. First, it could reflect a signal-to-noise phenomenon where higher doses result in a stronger N15 component (|S|), leading to improved focality and accuracy of source reconstruction. Second, it may indicate a genuine dose-dependent shift in N15 source location, as discussed further in Section 4.

Overall, the three inverse solvers exhibited strong agreement in terms of spatial concordance. They all identified similar significant nodes, provided consistent interpretations, and revealed comparable main effects of field type. Overall, eLORETA yielded the most divergent results, showing a broader distribution of N15 source activity relative to the TMS-induced electric fields, particularly in the frontal ipsilateral and contralateral regions.

### 3.3 The N15 source is state- and dose-dependent

Our secondary aim was to replicate and build on the results of prior work from our group (Beck et al., 2025). Specifically, we assessed if N15 |S| depends on motor state and/or dose, both in terms of |S|_P99.9_ and in terms of whole-cortex |S|.

**Fig. 5.**
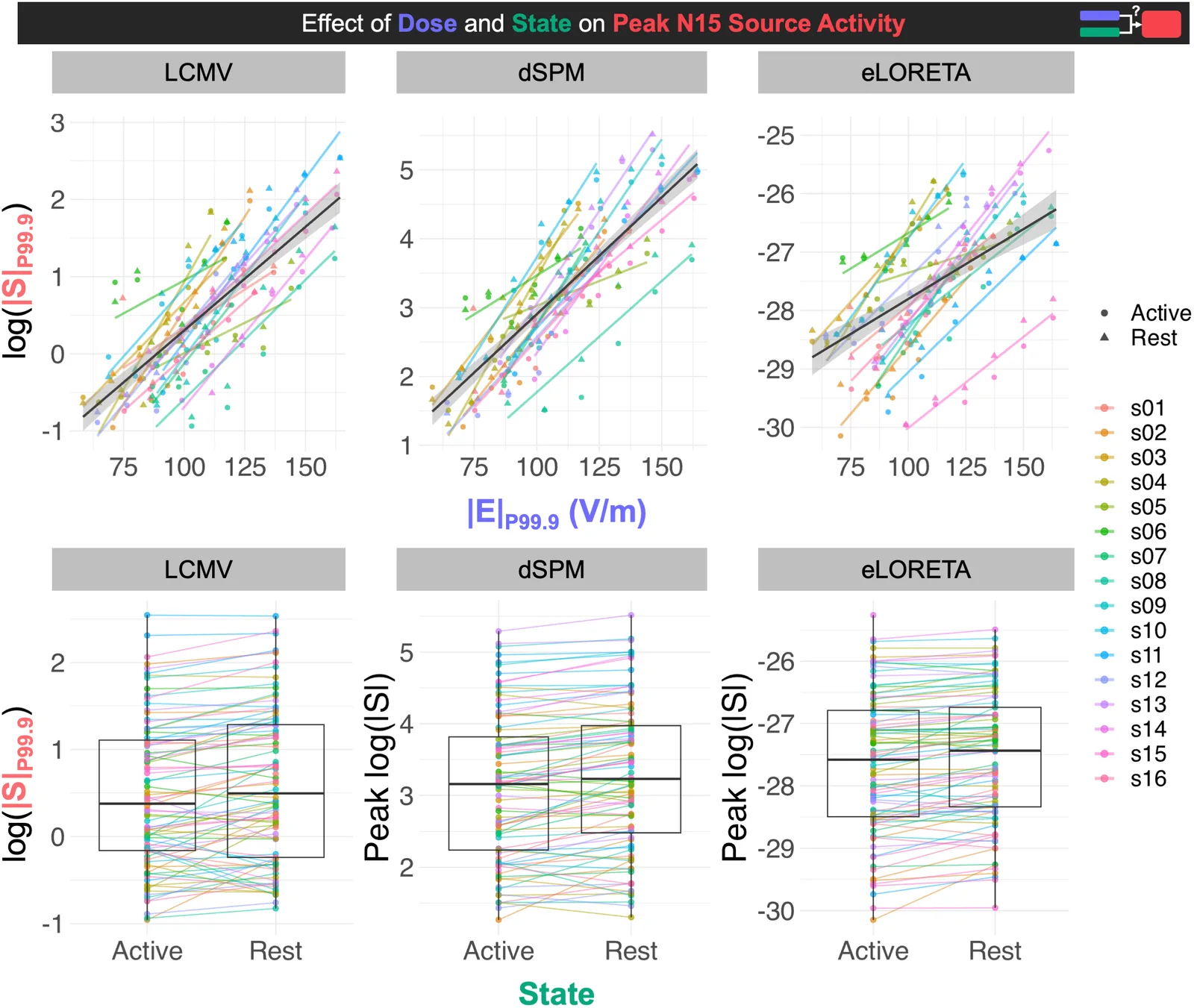
Effect of dose (|E|_P99.9_) and state on peak N15 magnitude (|S|_P99.9_). Higher doses result in greater N15 magnitude (upper row). N15 magnitude during rest is greater than during active motor state (lower row). Results are consistent across all three solvers. Colors relate to participant indices, going from s01 to s16.

Regarding peak source magnitude, our results concur with (Beck et al., 2025). State (all p < 0.05) and dose (all p < 0.001) yielded a significant main effect across all three solvers. Detailed linear mixed-model results are included in Supplementary Section S4 (Supplementary Tables S4.2–S4.4). Across all solvers, higher |E|_P99.9_ was associated to greater |S|_P99.9_ (all partial η^2^ > 0.79) (Fig. 5, upper panel). N15 |S|_P99.9_ elicited by TMS during rest was larger, compared to active, with effect sizes ranging from small (LCMV, η^2^ = 0.04) to medium (dSPM and eLORETA, η^2^ = 0.06).

The intraclass correlation (ICC) was high for all three solvers (ICC > 0.62) indicating that a high proportion of variance stemmed from between-participant differences. This was particularly true for eLORETA, with an ICC = 0.88. Also, the conditional R^2^ of eLORETA (0.94) exceeded that of the other solvers (0.88 [LCMV] and 0.90 [dSPM]), while its marginal R^2^ was comparatively lower (0.49 compared to 0.67 [LCMV] and 0.72 [dSPM]). Thus, eLORETA accounted for less variance through fixed effects, while the variance across individuals remained substantial.

The source maps concur with the spatial concordance analyses in that N15 originates from the left premotor and motor regions (Fig. 5, right panel), which are rostral to the TMS induced electric field (Fig. 3).

**Fig. 6.**
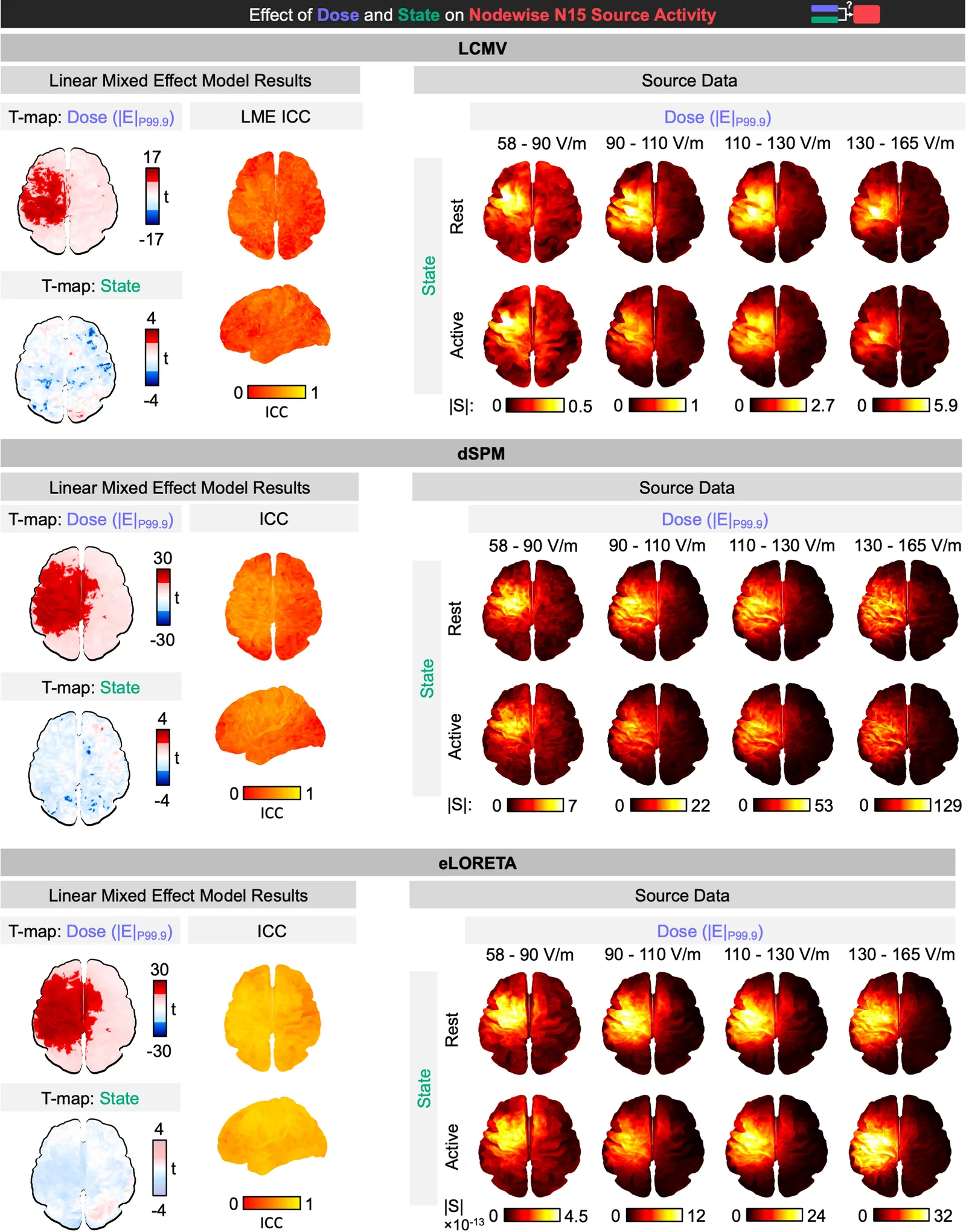
Effect of dose and state on nodewise N15 source magnitude (|S|). Per solver, for dose and state, the t-maps (left panel) show the significant nodes of the spatial clustering analyses. All solvers show a positive effect of dose on |S|, centered over the left premotor- and primary motor cortex. The middle panel shows the ICC, with between-subject variability being lowest for LCMV and highest for eLORETA. On the right, group-average source data are shown for four dose bins and both motor states.

For all three inverse solvers, a significant effect of dose on |S| was found, which seemed to be driven by a cluster centered over the left premotor cortex and M1. The effect of state was less pronounced on the whole-cortex level compared to peak data, with only some nodes in some solvers, mainly LCMV, surviving multiple comparison correction and the surviving nodes not being in accordance to the location of the actual source activity. Consistent with the |S|_P99.9_ analyses and particularly for eLORETA, the ICC values of the nodewise analyses were relatively high (Fig. 6, middle panel).

## 4 Discussion

Using anatomically detailed forward models and three inverse solvers, we have elucidated various facets of the N15 TEP peak generated from TMS over the motor cortex. We also reproduced prior findings on the source-level. Our discussion is structured into three sections. First, we discuss the origin of N15 (Section 4.1). Next, we discuss the state- and dose-dependence of N15 |S| (Section 4.2), followed by a performance evaluation of the three inverse solvers (Section 4.3).

### 4.1 N15 following primary motor cortex TMS originates from the ipsilateral (pre-)motor regions, with its precise origin possibly being dose-dependent

Our findings concur with prior research showing that the N15 peak following M1 stimulation arises from the regions rostral to the central sulcus, encompassing the ipsilateral M1 and premotor cortex (Esser et al., 2006; Litvak et al., 2007; Mäki & Ilmoniemi, 2010; Zazio et al., 2021). The N15 source field is partially distinct from the TMS electric field, which is primarily localized on the crowns of the pre- and post-central gyri (cf., Section 3.1).

If one cautiously interprets the TMS-induced electric field as a proxy for the directly activated neuronal populations (cf., Section 4.4), this spatial dissociation suggests that N15 does not represent a simple (re)activation of neurons first responding to the TMS-pulse within the first milliseconds post-stimulation (Lazzaro et al., 1998). Instead, these data support the interpretation that N15 reflects subsequent activity of at least partially distinct neuronal populations, potentially via cortico-thalamo-cortical or cortico-cortical loops (Farzan & Bortoletto, 2022; Li et al., 2017). If genuine, this spatial discrepancy may also help explain the observation that MEPs and N15 show distinct responses to motor state, as the former increases in an active motor state whereas the latter decreases (Beck et al., 2025).

Importantly, the interpretation that the N15 component stems from distinct sources than the ones being initially activated by TMS does not solely rely on the assumption that electric field exposure informs on cortical response to TMS. Converging evidence shows that TMS over M1 induces an initial, direct, cortical response localized to the precentral gyral crown or central sulcus with some work favoring more superficial sites (i.e., the crown) (Beck, Christiansen, et al., 2024; Bungert et al., 2017; Kataja et al., 2021; Laakso et al., 2018; Numssen et al., 2021; Nuyts et al., 2025; Siebner et al., 2022; Weise et al., 2020), regions that are both caudal to the estimated N15 source field and overlapping with the peak electric field magnitude.

At the same time, two considerations should temper interpretations. First, as already mentioned, electric field magnitude is not a one-to-one proxy for neuronal activation. Second, while the forward model captures tissue boundaries at the macro-level, it relies on group-estimate conductivity values. Uncertainty in these scalar conductivity values, and their discontinuity across tissue boundaries, may introduce additional uncertainty of the modeled field; however, for TMS, modeling work has shown that this affects field magnitude more than spatial distribution (Saturnino et al., 2019). In principle, systematic conductivity differences between the pre- and postcentral gyri and the more anterior brain areas might be able to systematically shift the E-field peaks. This possibility could be investigated in future sensitivity analyses. If so, the present findings would constitute one of the first experimental indications of this phenomenon, with implications for the validation and refinement of E-field models used to guide TMS targeting. We note, however, that this interpretation would require such distortions to be more substantial, spatially selective and consistent across participants than current modeling evidence on conductivity uncertainty generally suggests (Saturnino et al., 2019). It is also at odds with the results of the functional hotspot search which resulted in coil positions that maximized the calculated group-level E-field in the handknob area of the precentral gyrus. Finally, it does not account for the additional finding that the N15 source location shifted systematically with stimulation dose (Section 4.1), a pattern not obviously predicted by a static, conductivity-driven distortion of the electric field.

The impact of TMS dose might extend beyond N15 magnitude (Figs. 5 and 6), as it also affects its location and thus the primarily activated neurons (Fig. 4). This could reflect a genuine neurophysiological mechanism, although it could also be a signal-to-noise effect, or a combination of both. In support of this dose-dependent shift in N15 origin, plausibly via activations of new sources with higher thresholds, are previous TMS-fMRI studies, indicating that repetitive TMS over M1 at subthreshold intensities results in activation of non-targeted remote motor areas such as SMA-proper, rather than M1 (Bestmann et al., 2003, 2004). Also, Komssi et al. (2004) observed a dose-dependent shift in the N15 source location, elicited by TMS-EEG over left M1, albeit that this may have also represented a signal-to-noise effect.

### 4.2 N15 magnitude depends on motor state and TMS dose

Our results agree with previous sensor-level EEG analyses in that N15 following M1 stimulation depends on dose and state (Beck et al., 2025; Nuyts, Siebner, et al., 2026), and can be observed at intensities well-below the rMT (Beck et al., 2025; Komssi et al., 2007). Dose definitions based on rMT ± %MSO, absolute MSO and peak |E| yielded comparable model fits for peak N15 |S| in the current work (Section 3.1). The direct comparison of these three dose-related metrics further underscores their similarity within our experimental design (Supplementary Fig. S2.5), corroborating prior findings (Caulfield et al., 2021).

Nevertheless, these results should not be interpreted as evidence that the three dose definitions are interchangeable. The employed linear mixed-effects models included random intercepts per participant, thereby absorbing inter-individual differences in N15 amplitude. When comparing |E| and %MSO within discrete dose levels defined relative to RMT (rMT – 8% to rMT + 12%, Supplementary Fig. S2.6), |E| consistently yielded better model fits across all three inverse solvers, indicating that the added macro-anatomical information incorporated in current flow modeling can be advantageous when studying inter-individual differences.

### 4.3 LCMV, dSPM, and eLORETA produce convergent results

While an absence of a ground-truth made direct comparisons between the different inverse solvers unattainable, our study represents one of the first attempts to compare multiple inverse solvers in the context of TEPs. Overall, LCMV, dSPM and eLORETA mostly concurred, corroborating previous research performing comparisons in other EEG domains (Grech et al., 2008; Halder et al., 2019). However, LCMV and dSPM exhibited the most similar performance, both in terms of predicting peak- and whole-cortex N15 |S|, based on the marginal R^2^ and ICC values (Section 3.2). Conversely, eLORETA demonstrated smaller marginal R^2^ values and higher ICC values, indicating a poorer prediction of dose and state effects on |S|, as well as greater interindividual variability, at least for the investigated parameter space. This pattern was also observed when examining inter-individual differences in peak N15 |S| within discrete rMT ± %MSO dose levels, where both |E| and %MSO were more predictive of |S| for LCMV and dSPM than for eLORETA (Supplementary Fig. S2.6). Minimum-norm-estimation (Supplementary Section 2, Supplementary Fig. S2.2) yielded converging results in terms of peak |S|, but ICC values were higher and the localization of N15 on the whole-brain was not as clear.

Thus, these findings suggest that while different inverse solvers exhibit some variability in their sensitivity to dose and state effects, their overall performance in analyzing TEPs seems broadly consistent. Importantly, this convergence is, to an extent, likely attributable to the shared preprocessing pipeline and common forward model, including identical FEM-based head models, source space definitions, and covariance estimation procedures. These common choices may have introduced systematic biases across solvers. Future work varying these underlying attributes would further inform on the sensitivity of the various solvers to these parameters.

### 4.4 Limitations and future recommendations

Several limitations warrant consideration and point toward directions for future work. First, our comparison between electric field distributions and source-localized activity assumes that the cortical region experiencing the highest electric field magnitude corresponds to the site of effective stimulation. This is an approximation, as neuronal activation depends on many additional factors such as field orientation, cytoarchitecture, and membrane excitability. Nevertheless, prior work indicates that TMS over M1 preferentially activates the crown or posterior lip of the precentral gyrus (Aberra et al., 2020; Bungert et al., 2017; Laakso et al., 2018; Numssen et al., 2021; Siebner et al., 2022; Weise et al., 2020), regions that, in our data, coincide with the loci of maximal electric field strength and are located caudal to the N15 source. Thus, even if the peak electric field does not entirely coincide with the true site of effective stimulation, currently available evidence supports the interpretation that the N15 source is situated predominantly rostral to the stimulated cortical region. Relatedly, as discussed in Section 4.1, we cannot exclude that conductivity-related distortions of the modeled electric field contribute to part of this offset. Rather than solely a limitation, this raises the possibility that TMS-EEG source localization could serve as an in vivo complement to existing E-field model validation approaches, which have so far relied primarily on intracranial recordings which have their limitations (Puonti, Saturnino, et al., 2020).

Second, we focused on the magnitudes of the N15 source- and the TMS-induced electric fields, without considering their orientation relative to the cortical surface. The N15 source field is thought to predominantly reflect synchronized postsynaptic dendritic currents in pyramidal neurons (Kirschstein & Köhling, 2009). Conversely, TMS activation preferentially occurs on axons (Aberra et al., 2020; Siebner et al., 2022). Consequently, the orientations of the EEG source- and the TMS electric field are not expected to be strongly coupled. Nevertheless, future work could extend our approach by explicitly incorporating orientation-specific field properties.

Third, in nine participants, neuronavigation data were corrupt, necessitating the interpolation of TMS coil positions in these individuals. Considering this, the observation that a dose definition using electric field magnitude (which relies on neuronavigation data) performed similarly as dose definitions operating independently of neuronavigation data (i.e., %MSO and rMT-derived) is reassuring. The control analyses reported in Supplementary Materials 3 provide further support for the validity of our interpolation approach: leave-one-out validation in participants with intact neuronavigation data revealed only negligible differences in electric field distribution between the genuine and interpolated data. Specifically, whereas the average distance between the genuine and the interpolated coil positions was 7.23 mm (range: 3.65–12.05 mm), the average difference between the grey matter centroids receiving electric fields above the 99.9th percentile was only 2.88 mm (range: 0.65 – 6.94 mm). Moreover, the dose-dependent shift in N15 source activation was qualitatively preserved within the subgroup with intact neuronavigation data, with |E|–|S| maps closely resembling those presented in Figure 4.

Fourth, while our forward models are among the anatomically most accurate to-date, the conductivity values are based on reference values. While relatively less important for TMS, erroneous conductivity values can affect the accuracy of EEG source reconstruction (Vorwerk et al., 2019).

Fifth and more generally, while our sample size aligns with previous TMS-EEG studies, it was not determined through formal sample size calculations due to a lack of comparable studies.

Finally, our findings should be interpreted considering a general limitation inherent to TMS-EEG: TEPs, including the N15 component, reflect not only direct cortical activation but may also be affected by residual muscular artefacts and multisensory contributions, if not dealt with properly. With respect to muscular artefacts, we adopted a conservative strategy based on stringent visual inspection and exclusion of participants with any signs of contamination. The resulting sensor-level data (Fig. 2, and Supplementary Figs. S2.3 and S2.4) show spatially distributed, dipolar topographies rather than the focal, lateralized patterns typically associated with muscle activity, supporting a predominantly neural origin of the observed effects. Regarding multisensory contributions, auditory and somatosensory co-activation are difficult to fully eliminate in TMS-EEG paradigms, albeit we adhered to the current standard of modified earplugs playing pink noise with recorded TMS clicks. The very early latency of the N15 (~15 ms) makes a dominant contribution from such inputs less likely compared to later components (Conde et al., 2019).

## 5 Conclusion

Using anatomically detailed forward models and three inverse solvers, we demonstrate that the cortical source underlying the N15 TEP peak following M1 stimulation is spatially distinct from the TMS-induced electric field. While the electric field peaked in the crowns of the pre- and post-central gyri, the N15 sources localize to more rostral regions and vary systematically with stimulation dose. Beyond its neurophysiological implications, this spatial offset may inform future efforts to validate and refine individualized TMS E-field models, with potential relevance for improving the spatial precision of rTMS-based interventions.

Our method provides a framework for investigating how TMS-evoked cortical activity relates to the underlying electric field. Future work can use this framework to examine additional TEP components and to assess how factors such as brain state, stimulation intensity, and clinical conditions modulate the coupling between TMS electric field and EEG-derived source activity. Recent studies capturing cortical responses within milliseconds of motor-cortex stimulation (Beck, Christiansen, et al., 2024; Nuyts et al., 2025) underscore the importance of this line of inquiry, as such immediate TEPs are expected to align closely with the spatial profile of the induced electric field.

## Supplementary Material

Supplementary Material

## Data Availability

The data underlying this article are not publicly available due to ethical and regulatory constraints. Access may be granted to qualified researchers upon submission of a motivated request, subject to (i) the establishment of a formal data-sharing agreement, (ii) approval from the requesting researcher’s local ethics committee, and (iii) submission and approval of a detailed project outline describing the planned analyses and intended use of the data. Fulfilment of these conditions is required prior to any data sharing. The analyses code are available from the corresponding author upon reasonable and justified request.
