# Supplementary material for: Sources of the N15 TMS-evoked potential following motor cortex stimulation localize rostrals to TMS-induced electric fields and depend on dose
Source: Imaging Neurosci (Camb). 2026 Sep 10;4:IMAG.a.1356. doi: 10.1162/IMAG.a.1356 (PMC13563533; doi:10.1162/IMAG.a.1356)
Supplement: Supplementary file 1 [file IMAG.a.1356_supp.pdf]

SUPPLEMENTARY MATERIAL - SOURCES OF THE N15 TMS-EVOKED POTENTIAL FOLLOWING MOTOR CORTEX STIMULATION LOCALIZE ROSTRAL TO TMS-INDUCED ELECTRIC FIELDS AND DEPEND ON DOSE

Sybren Van Hoornweder; Mikkel M. Beck; Jesper D. Nielsen; Leo Tomasevic; Raf L.J. Meesen; Hartwig R. Siebner; Axel Thielscher

S1 TISSUE CONDUCTIVITIES

Table S1.1. Conductivities used for TMS and EEG simulations.

Tissue Name	Value (S/m)	Reference
White Matter	0.126	[72]
Gray Matter	0.275	[72]
CSF	1.654	[72]
Scalp	0.465	[72]
Eye balls	0.5	[73]
Compact Bone	0.008	[73]
Spongy Bone	0.025	[73]
Blood	0.6	[74]
Muscle	0.16	[74]

S2 Supplementary Figures

Interpolated channels

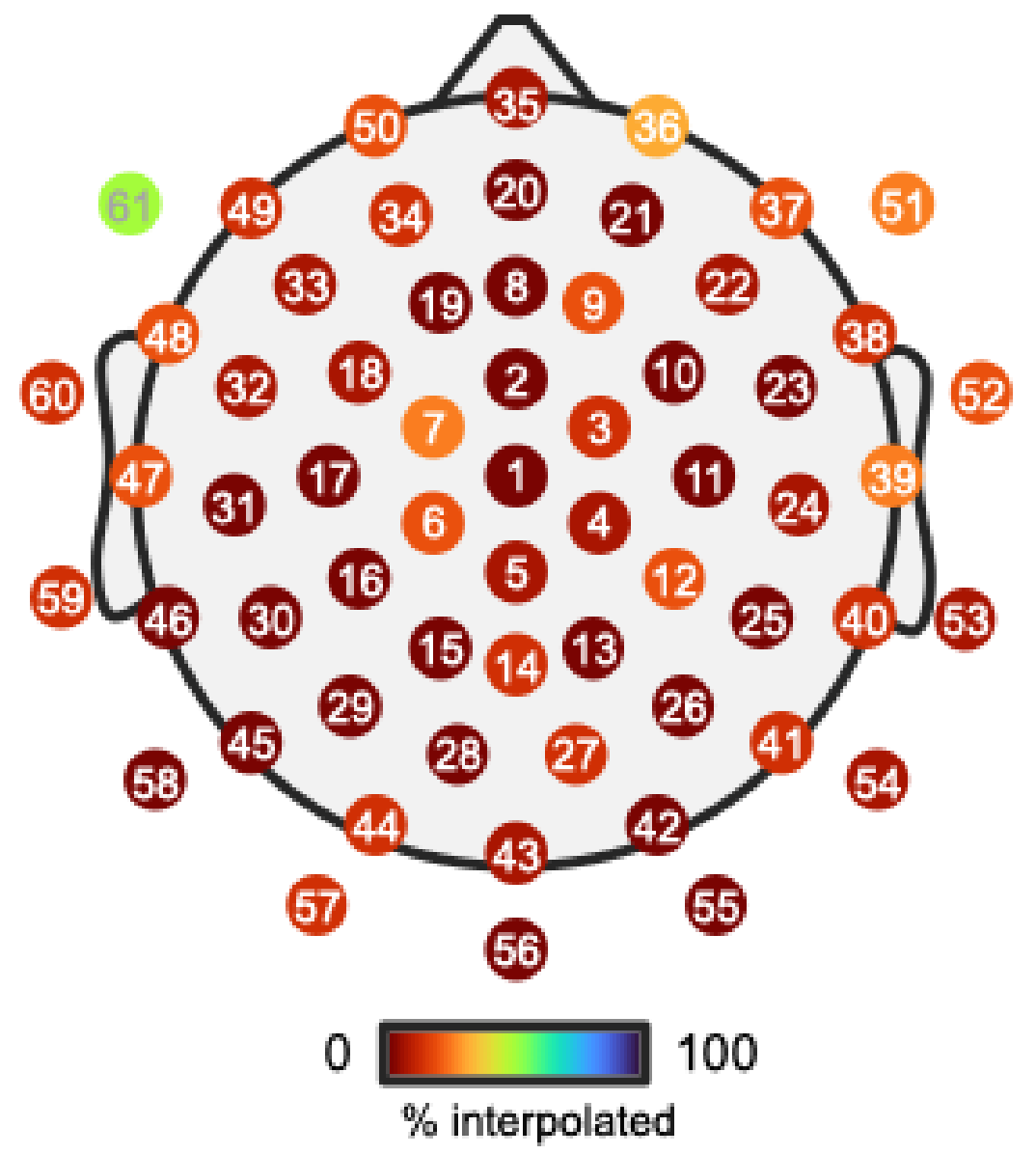

Rank	Channel	% Interpolated
1	61	50%
2	36	69%
3	7	75%
4	39	75%
5	51	75%
6	6	81%
7	9	81%
8	12	81%
9	37	81%
10	47	81%
11	48	81%
12	50	81%
13	52	81%
14	3	88%
15	14	88%
16	27	88%
17	34	88%
18	38	88%

Rank	Channel	% Interpolated
19	40	88%
20	41	88%
21	44	88%
22	49	88%
23	57	88%
24	59	88%
25	60	88%
26	4	94%
27	5	94%
28	18	94%
29	22	94%
30	24	94%
31	32	94%
32	33	94%
33	35	94%
34	43	94%
35	53	94%
36	54	94%

Figure S2.1. Visualization of the percentage of times channels were interpolated across participants. The left topographic plot visualizes how many times each channel was interpolated. The right table shows the exact number of times a channel was interpolated, with channels that were never interpolated being excluded.

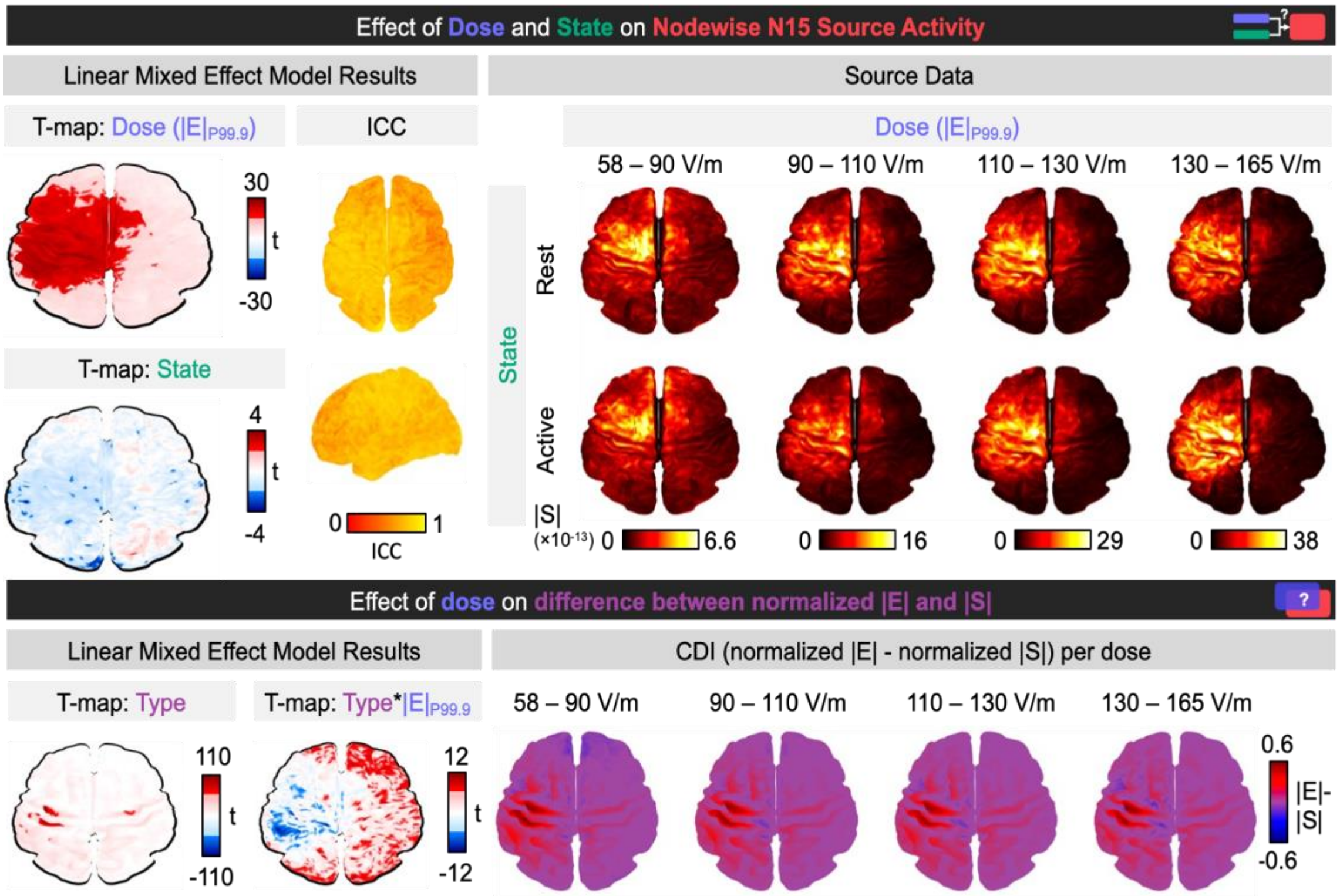

Figure S2.2. Minimum Norm Estimation. Source modeling results using minimum norm estimation as inverse solver. The magnitude-related analyses (upper panel) converge with the three other inverse solvers for the most part, albeit that the ICC was higher for minimum norm estimation. Results from the spatial concordance analyses (lower panel) converge with the other solvers in that normalized |E| was higher than normalized |S| in the pre- and post-central gyral crowns. Notably, |S| was not significantly larger in the rostral regions then |E|, disagreeing with the other solvers. Likely, this was due to |S| being less clearly localized. As the results of MNE differed the most compared to the other solvers, we also tested different depth-weighing factors of 0.8 and 5, next to 2. These weighting factors did not improve concordance with the other solvers.

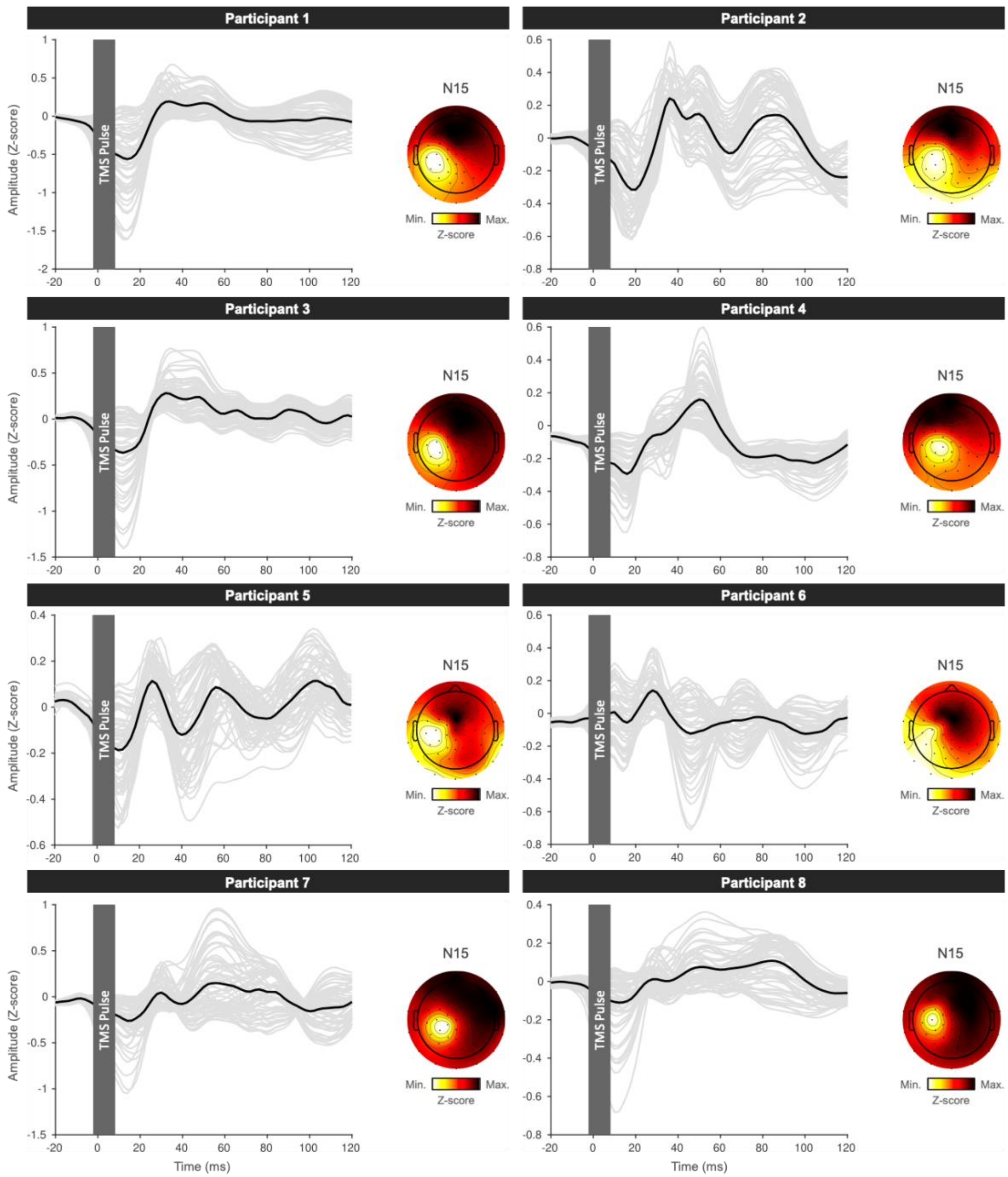

Figure S2.3. TMS-evoked potentials for participants 1 - 8. Per participant, a butterfly plot displays the grand-average TEP across channels (black line = average, grey lines = single channels). The corresponding N15 scalp topography (~16 ms) is also shown. Prior to averaging, data per state and condition were Z-score normalized to prevent higher-intensity TMS

conditions from disproportionately influencing the grand averages. Group-level state- and dose-specific effects are presented in **Figure 2** of the main text.

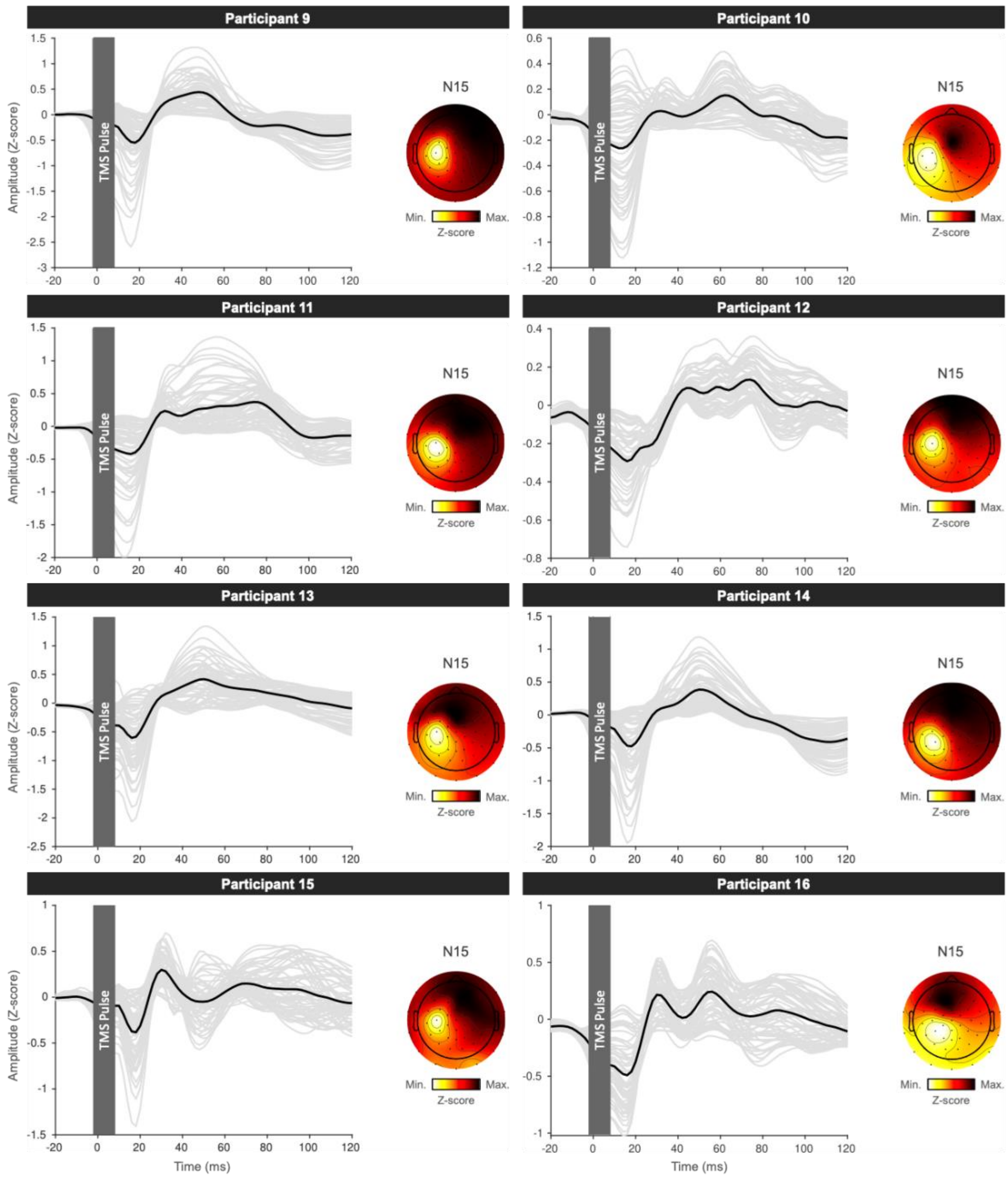

Figure S2.4. TMS-evoked potentials for participants 9 - 16. Per participant, a butterfly plot displays the grand-average TEP across channels (black line = average, grey lines = single

channels). The corresponding N15 scalp topography (~16 ms) is also shown. Prior to averaging, data per state and condition were Z-score normalized to prevent higher-intensity TMS conditions from disproportionately influencing the grand averages. Group-level state- and dose-specific effects are presented in **Figure 2** of the main text.

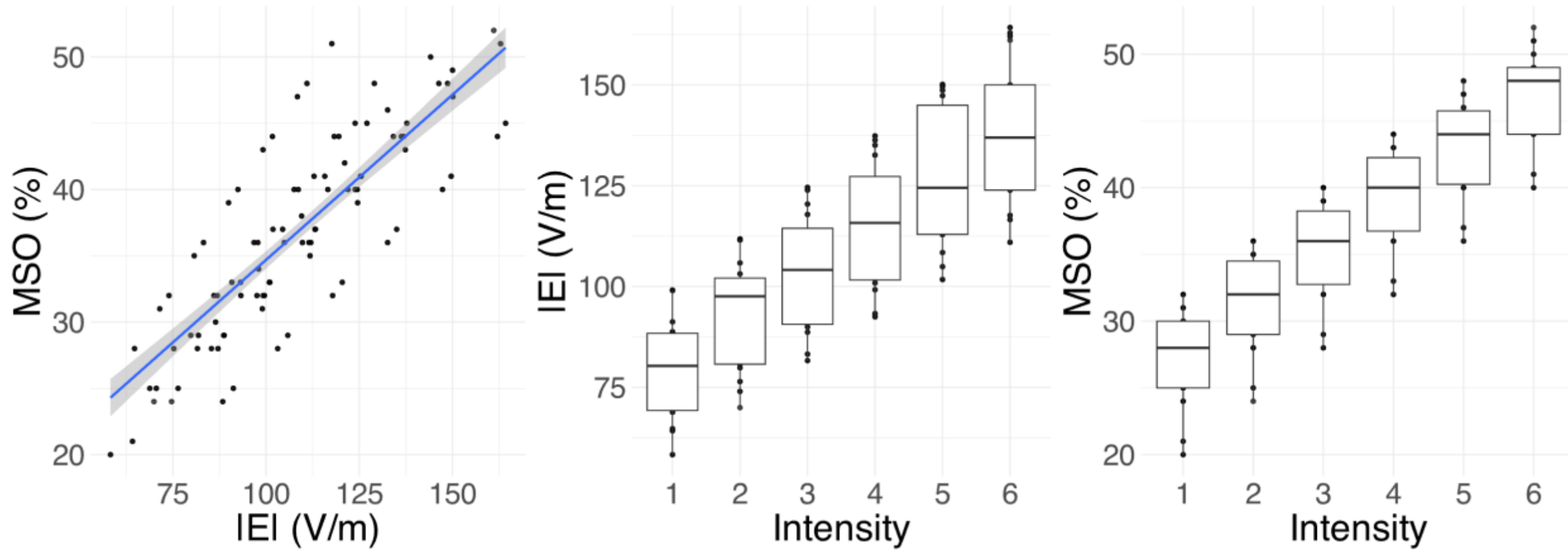

Figure S2.5. Relationship between |E|P99.9, %MSO and rMT-derived intensity. The relationship between the three different approximations of dose. The left panel shows the relationship between |E|P99.9 and %MSO, with the associated linear mixed effects model (|E|P99.9 ~ %MSO + (1| participant) obtaining a marginal R2marginal of 0.71. The middle panel shows the relationship between the rMT-derived intensity, ranging from 1 (rMT – 8%MSO) to 6 (rMT + 12%MSO) in 4% increments and |E|P99.9, with the associated linear mixed effects model (|E|P99.9 ~ Intensity + (1| participant) obtaining a marginal R2marginal of 0.63. The right panel shows the relationship between the rMT-derived intensity, ranging from 1 (rMT – 8%MSO) to 6 (rMT + 12%MSO) in 4% increments and %MSO, with the associated linear mixed effects model (%MSO ~ Intensity + (1|participant) obtaining a marginal R2marginal of 0.97. As to be expected, all three models, yielded an R2condition = 0.99.

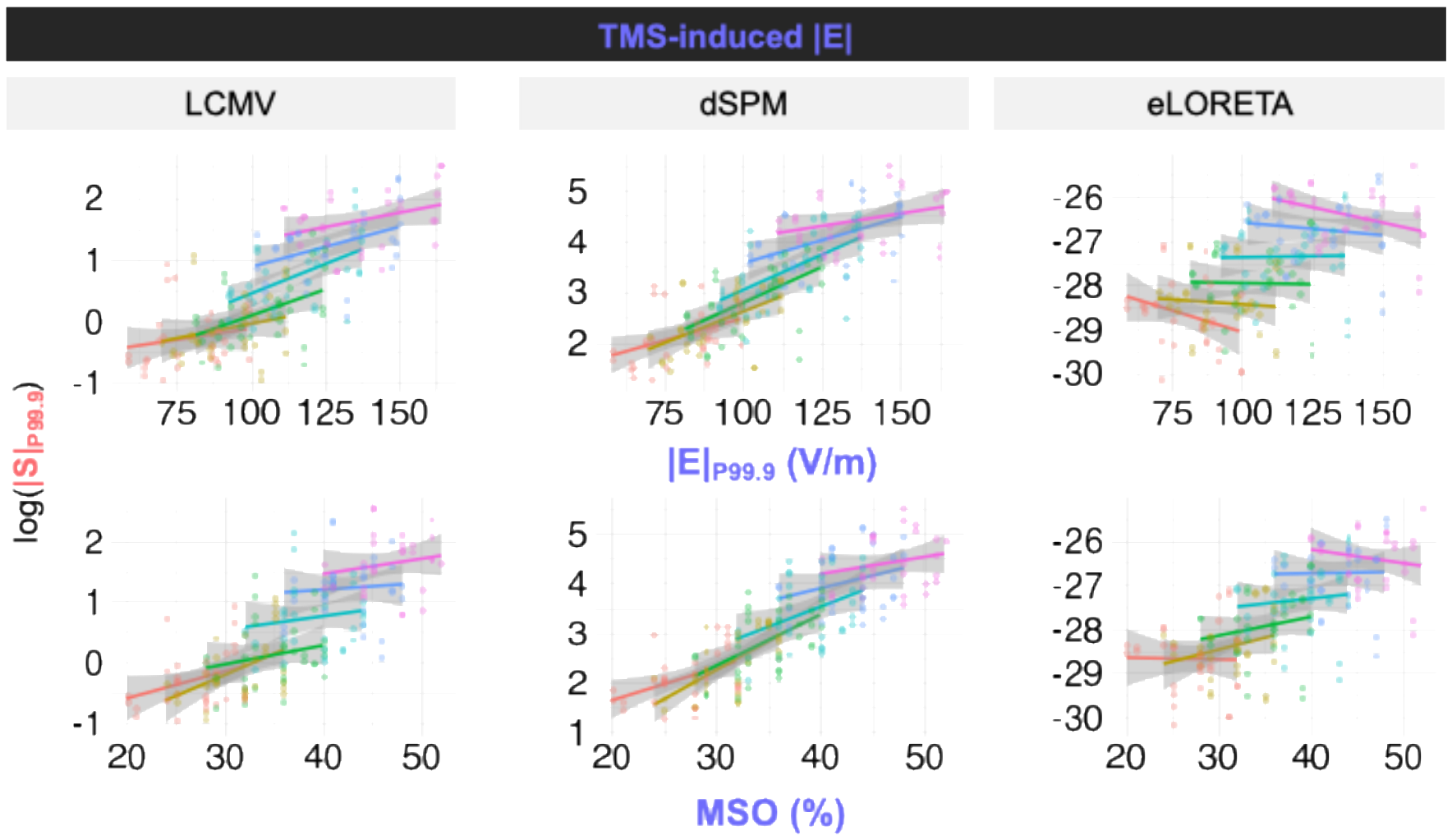

	LCMV		dSPM		eLORETA	
**Dose**	**%MSO**	**$\|E\|_{P99.9}$**	**%MSO**	**$\|E\|_{P99.9}$**	**%MSO**	**$\|E\|_{P99.9}$**
**R^2_m**	0.06 ± 0.02	0.16 ± 0.12	0.20 ± 0.13	0.25 ± 0.11	0.03 ± 0.02	0.04 ± 0.03
**AIC**	30.82 ± 8.81	28.79 ± 9.73	30.60 ± 8.70	28.21 ± 11.69	38.37 ± 11.95	37.75 ± 12.67
**BIC**	39.35 ± 8.95	37.32 ± 9.84	39.14 ± 8.83	36.75 ± 11.84	46.91 ± 12.07	46.28 ± 12.81

Figure S2.6. Comparison of two dose definitions (%MSO and $|E|_{P99.9}$) within each level of stimulation intensity, defined as rMT ± %MSO. In the upper figure, per inverse solver, the linear fits for per rMT ± %MSO dose definition are shown using |E| (upper row) and MSO (%) (lower row). In the lower table, the average model fits for the same linear mixed effects model as in the main text (cf., Figure 3) are shown per inverse solver and dose definition across the six discrete levels of rMT ± %MSO. Approximating dose via |E| consistently resulted in better more than fits than using %MSO, implying that |E| provides additional information beyond what %MSO delivers.

S3 Neuronavigation Data Interpolation: Control Analyses

Below, we present a series of control analyses evaluating the validity and robustness of the coil position interpolation approach.

First, **Figure S3.1** compares the induced electric field magnitudes in grey matter between participants with accurate neuronavigation data and those with corrupted data, for whom coil positions were reconstructed using group-average interpolation.

Second, **Figure S3.2** quantifies the accuracy of this interpolation procedure using a leave-one-out validation approach in participants with originally accurate neuronavigation data.

Third, **Figure S3.3.** shows the dose-dependent source E-field relationship of only the participants with accurate, non-interpolated, neuronavigation data, consistent with Figure 4 of the main text.

Finally, **Figure S3.4** assesses whether the main dose-response effects on TMS-evoked potentials are preserved when stratifying the dataset by interpolated versus non-interpolated coil position data.

Overall, these control analyses confirm that our interpolation approach preserved the broad |E| distribution.

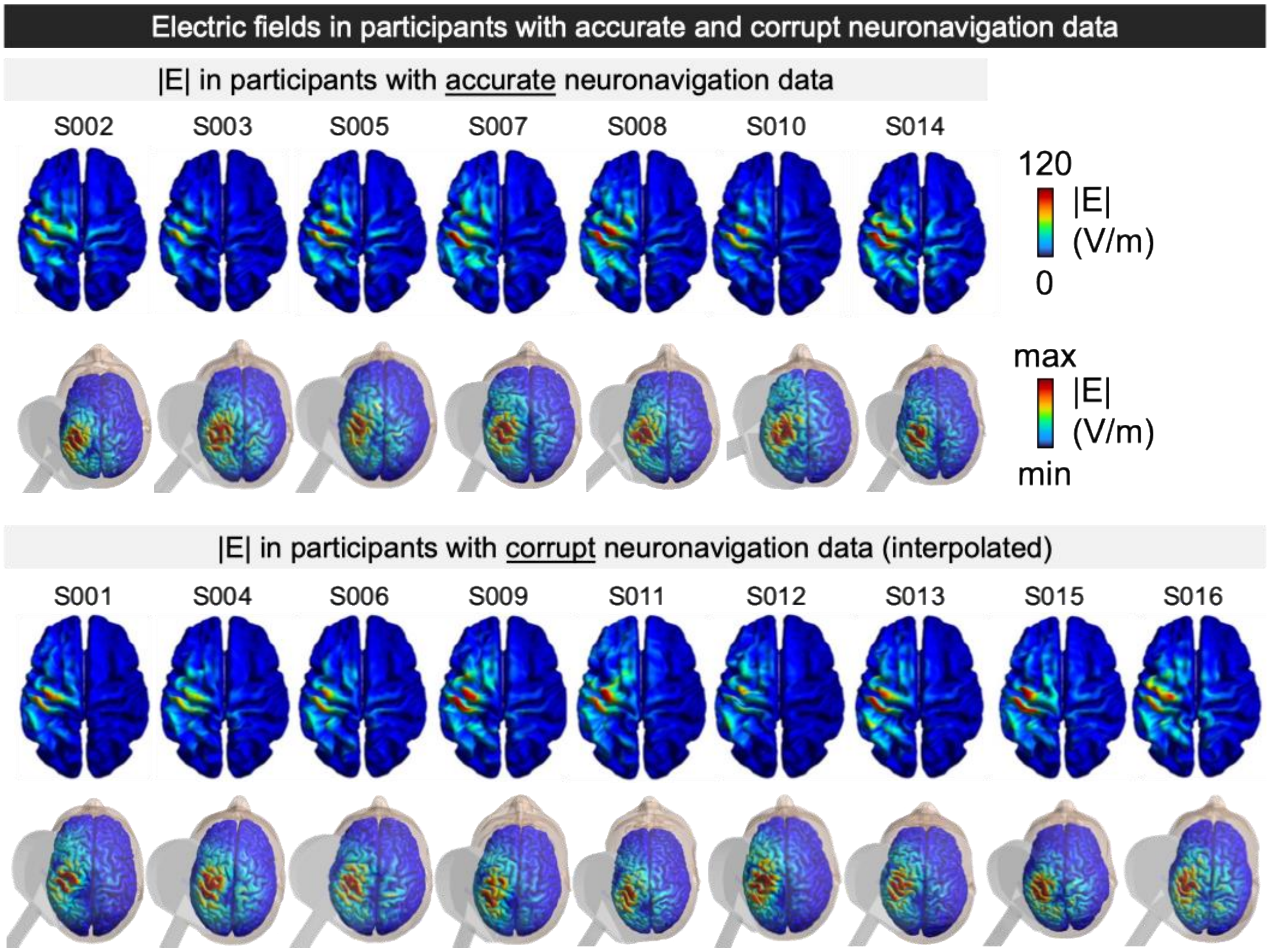

Figure S3.1. Electric field magnitude induced by TMS in participants with accurate and corrupt neuronavigation data at the rMT intensity. In the latter group, the coil position was interpolated based on the group-average coil position of the accurate neuronavigation group. The used scaling for the lower figures is identical to the scaling used in the top figures.

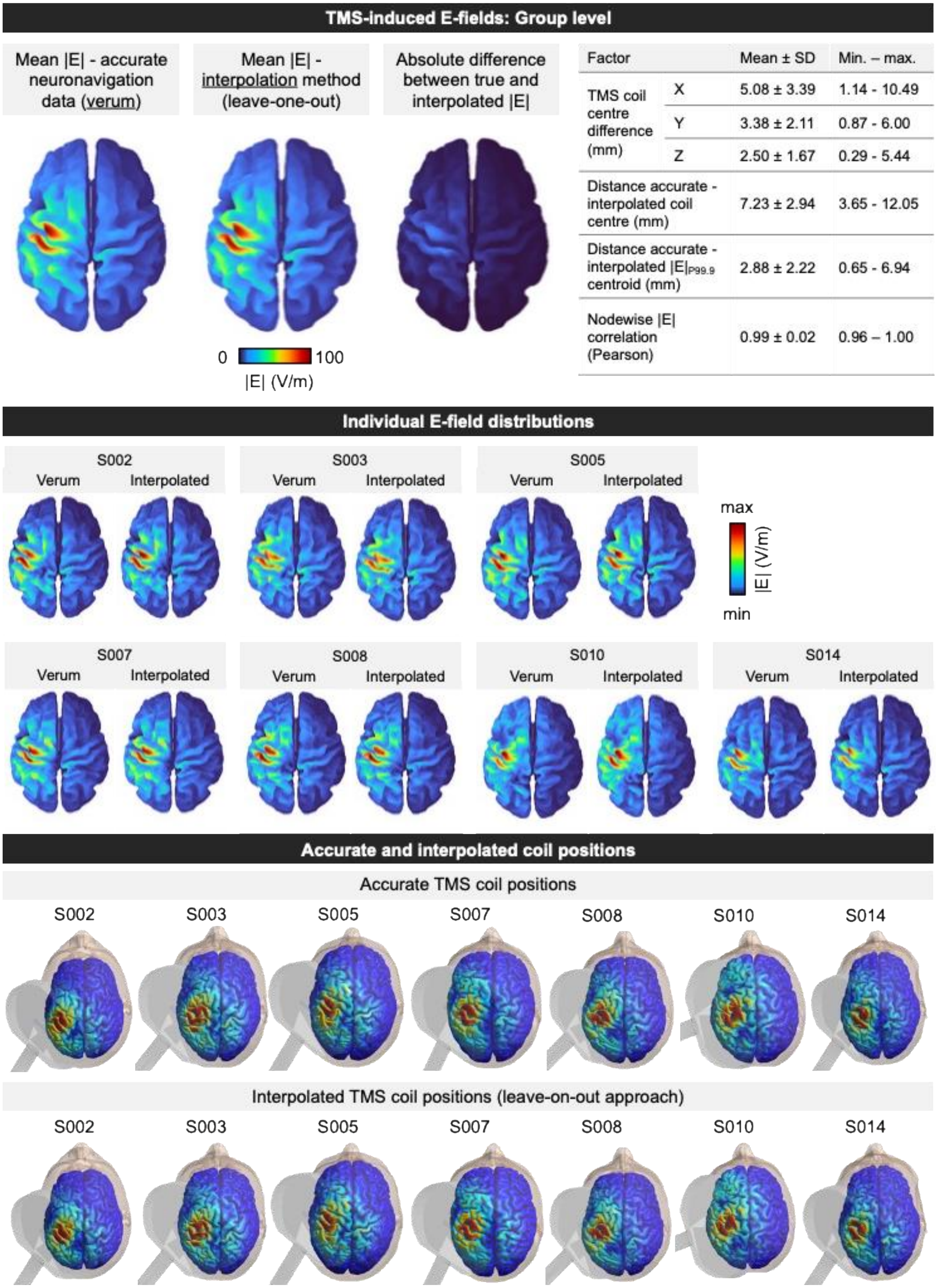

Factor		Mean ± SD	Min. – max.
TMS coil centre difference (mm)	X	5.08 ± 3.39	1.14 - 10.49
	Y	3.38 ± 2.11	0.87 - 6.00
	Z	2.50 ± 1.67	0.29 - 5.44
Distance accurate - interpolated coil centre (mm)		7.23 ± 2.94	3.65 - 12.05
Distance accurate - interpolated $\|E\|_{P99.9}$ centroid (mm)		2.88 ± 2.22	0.65 - 6.94
Nodewise |E| correlation (Pearson)		0.99 ± 0.02	0.96 – 1.00

Figure S3.2. Leave-one-out control analyses for non-interpolated (accurate) neuronavigation data.

Upper panel. Mean electric field magnitude (|E|) in fsaverage space for participants with accurate neuronavigation coil positions (left), the same data reconstructed using a leave-one-out interpolation approach (middle), and the difference between both (right). In the leave-one-out procedure, coil positions from six participants were used to estimate the coil position of the seventh, mirroring the reconstruction approach applied to participants with corrupted data. Difference maps indicate negligible discrepancies between actual and interpolated electric field distributions. This is further corroborated by the table on the right, showing minor differences in TMS coil position, with the Euclidean distance between the accurate and interpolated coil positions being 7.23 mm, alongside high correlations between nodewise |E| values in the interpolated vs. accurate current flow simulations (mean $r = 0.99$) and a 2.88 mm difference (Euclidean distance) between the peak |E| centroids of the interpolated and accurate models, with the centroids defined by all nodes exceeding the 99.9th percentile |E|.

Middle panel. Visualization of electric field simulations in fsaverage space for the accurate and interpolated (leave-one-out approach) positions for the sample of participants which had neuronavigation data. Highly similar current flow models were obtained for both procedures.

Lower panel. Visualization of coil position relative to the scalp and the resulting electric field magnitude across subjects in subject-space. For participants with accurate neuronavigation data, the top row shows original neuronavigation-based coil positions and corresponding electric fields, whereas the bottom row shows results from the leave-one-out control analysis.

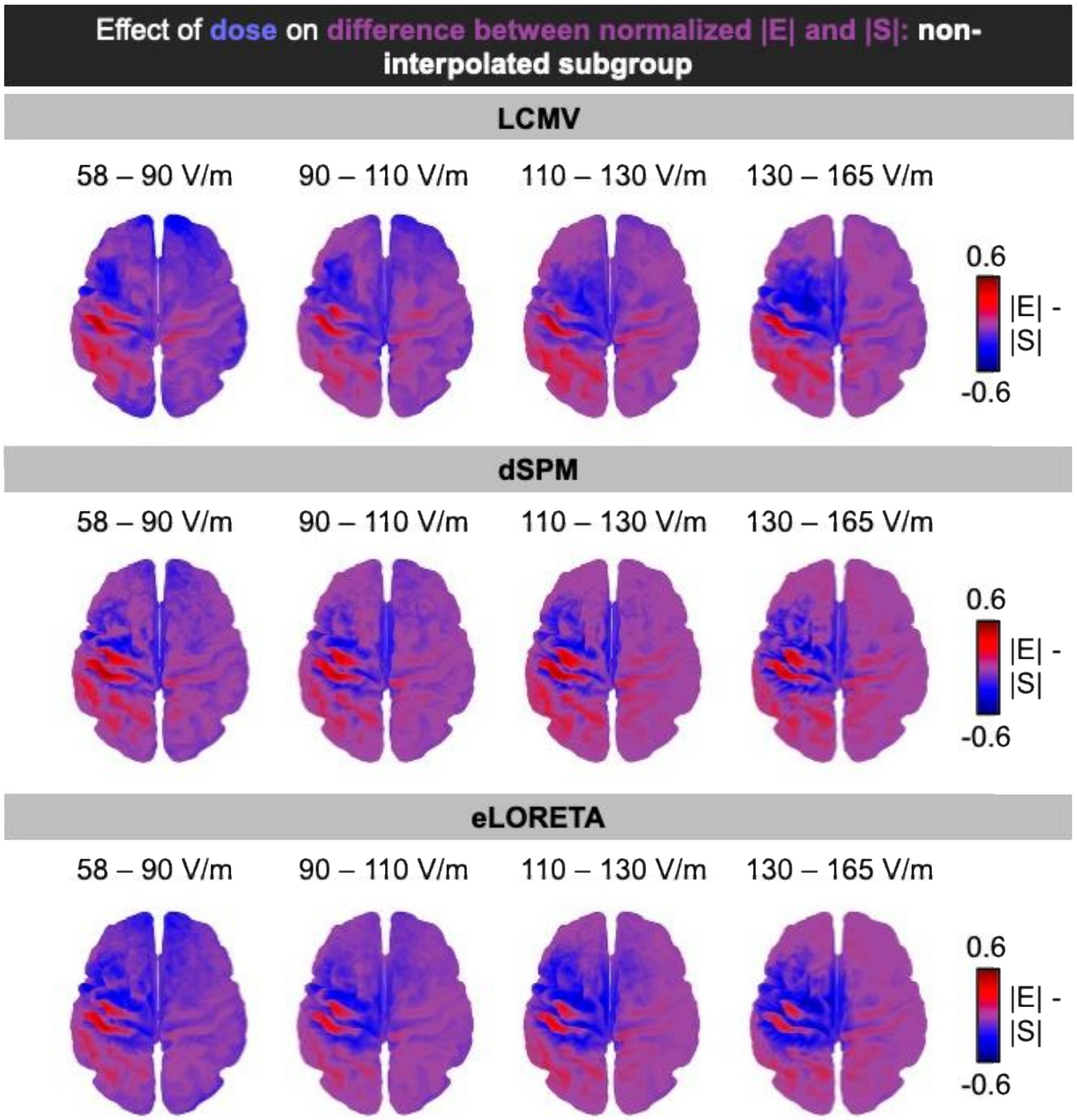

Figure S3.3. Spatial concordance between the TMS electric field (|E|) and N15 source field (|S|), per inverse solver, with only participant with accurate neuronavigation data included (n = 7). This figure largely resembles the right panel of Figure 4, main text, as it shows the normalized |E| - |S| difference maps. Consistent with the main text, |E| is larger in the pre- and post-central gyral crowns, while |S| is larger in the premotor regions. Higher doses reduce the relative difference between |E| and |S| in the crown of the precentral gyrus.

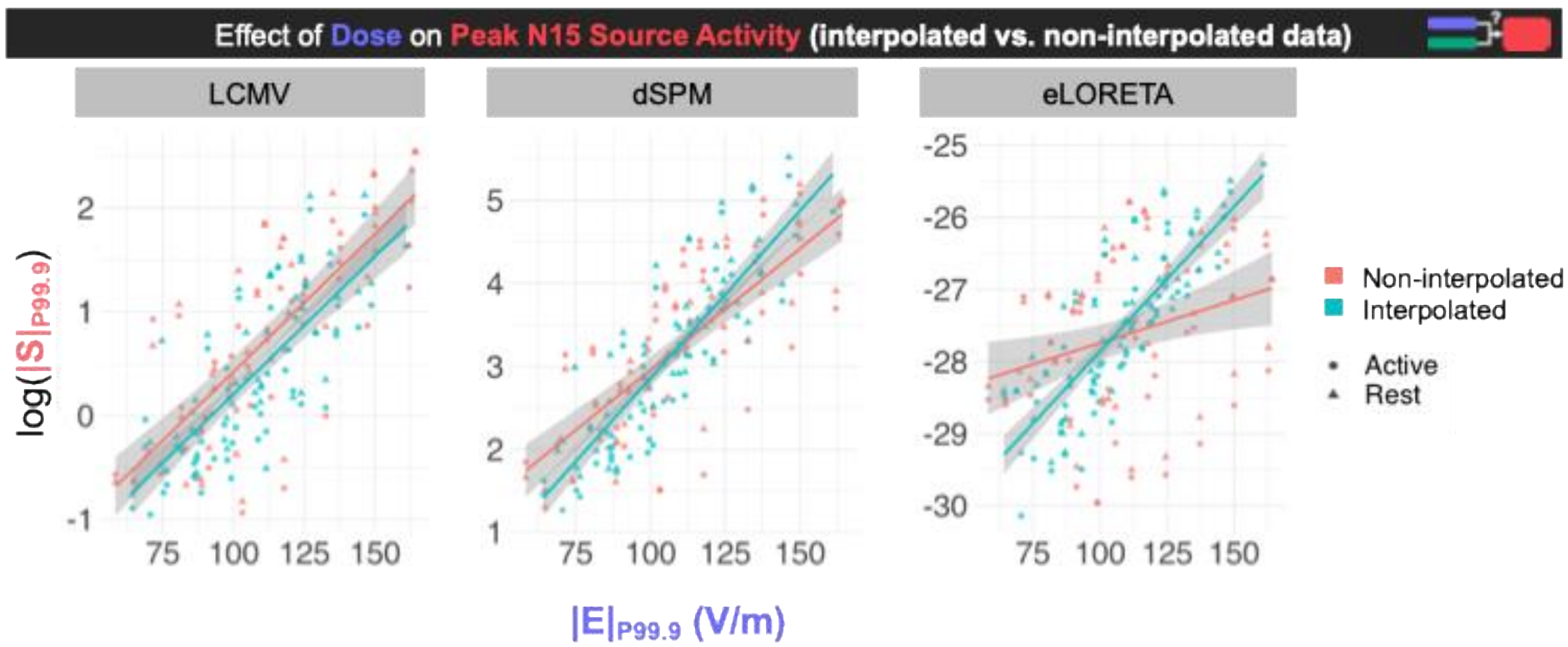

Figure S3.4. Effect of dose ($|E|_{P99.9}$) on peak N15 magnitude ($|S|_{P99.9}$), differentiating between participants with interpolated and non-interpolated TMS coil position data. Consistent with Figure 4 (main text), higher doses result in greater N15 magnitude across both groups (upper row). N15 magnitude during rest is greater than during active motor state (lower row). The statistical models presented in Section 3.3. were also repeated for both subsamples. For both the interpolated and non-interpolated data, dose effects are in line with the analyses presented in the main text, with a significant effect of dose ($p < 0.001$) across all solvers.

S4 Linear Mixed Effects Results – Model Tables

Table S4.2. Effect of dose ($|E|_{P99.9}$) and state on $|S_{LCMV}|_{P99.9}$

	log($\|S_{LCMV}\|_{P99.9}$)			
Predictors	*Estimates*	*CI*	*p*	*partial η²*
(Intercept)	-3.21	-3.59 – -2.84	**<0.001**	
Dose ($\|E\|_{P99.9}$)	0.03	0.03 – 0.04	**<0.001**	0.79
State [Rest]	0.13	0.03 – 0.24	**0.012**	0.04
**Random Effects**				
σ^2	0.13			
$\tau_{00\ id}$	0.22			
ICC	0.62			
N id	16			
Observations	184			
Marginal R^2 / Conditional R^2	0.670 / 0.876			

Table S4.3. Effect of dose ($|E|_{P99.9}$) and state on $|S_{dSPM}|_{P99.9}$

	log($\|S_{dSPM}\|_{P99.9}$)			
Predictors	*Estimates*	*CI*	*p*	*partial η²*
(Intercept)	-1.32	-1.71 – -0.93	**<0.001**	
Dose ($\|E\|_{P99.9}$)	0.04	0.04 – 0.04	**<0.001**	0.85

State [Rest]	0.18	0.07 – 0.28	**0.001**	0.06
**Random Effects**				
σ^2	0.14			
$\tau_{00\ id}$	0.26			
ICC	0.66			
N $_{id}$	16			
Observations	184			
Marginal R^2 / Conditional R^2	0.721 / 0.904			

Table S4.4. Effect of dose ($|E|_{P99.9}$) and state on $|S_{eLORETA}|_{P99.9}$

	**log($\|S_{eLORETA}\|_{P99.9}$)**			
Predictors	*Estimates*	*CI*	*p*	*partial η^2*
(Intercept)	-32.03	-32.58 – -31.48	**<0.001**	
Dose ($\|E\|_{P99.9}$)	0.04	0.04 – 0.04	**<0.001**	0.84
State [Rest]	0.16	0.06 – 0.26	**0.002**	0.06
**Random Effects**				
σ^2	0.12			
$\tau_{00\ id}$	0.90			
ICC	0.88			
N $_{id}$	16			
Observations	184			

Marginal R^2 / Conditional R^2	0.489 / 0.939
